# Circulating Microbial Metabolites Predict Tumor Relapse and Chemotherapy Efficacy in Nasopharyngeal Carcinoma

**DOI:** 10.1002/mco2.70687

**Published:** 2026-03-18

**Authors:** Jun‐Yan Li, Yao Yao, Xi‐Rong Tan, Nan Si, Wei Jiang, Ying‐Qi Lu, Jia‐Hao Dai, Tian‐Tian Yu, Hao‐Cheng Hu, Yu‐Fei Duan, Sen‐Yu Feng, Sai‐Wei Huang, Ye‐Lin Liang, Sha Gong, Na Liu, Yu‐Min Hu, Ying‐Qing Li

**Affiliations:** ^1^ State Key Laboratory of Oncology in South China Guangdong Key Laboratory of Nasopharyngeal Carcinoma Diagnosis and Therapy Sun Yat‐sen University Cancer Center Guangzhou People's Republic of China; ^2^ Department of Pathology Sun Yat‐sen University Cancer Center Guangzhou People's Republic of China; ^3^ Department of Experimental Research Sun Yat‐sen University Cancer Center Guangzhou People's Republic of China; ^4^ Department of Molecular Diagnosis Sun Yat‐sen University Cancer Center Guangzhou People's Republic of China; ^5^ Department of Radiation Oncology Affiliated Hospital of Guilin Medical University Guilin People's Republic of China; ^6^ Key Laboratory of Oncology (Guilin Medical University), Education Department of Guangxi Zhuang Autonomous Region Guilin People's Republic of China; ^7^ Department of Radiation Oncology Sun Yat‐sen University Cancer Center Guangzhou People's Republic of China; ^8^ Metabolomics Research Center Zhongshan School of Medicine Sun Yat‐sen University Guangzhou People's Republic of China; ^9^ School of Medicine Shenzhen Campus of Sun Yat‐Sen University Shenzhen People's Republic of China; ^10^ Department of Outpatient, State Key Laboratory of Oncology in South China, Guangdong Key Laboratory of Nasopharyngeal Carcinoma Diagnosis and Therapy Sun Yat‐sen University Cancer Center Guangzhou People's Republic of China

**Keywords:** circulating biomarker, metabolite, microbiota, nasopharyngeal carcinoma

## Abstract

The value of microbial metabolites in prognosis and treatment response prediction in patients with nasopharyngeal carcinoma (NPC) remains unclear. Here, through the untargeted metabolomic analysis of plasma in 48 paired NPC patients with or without tumor relapse, we identified distinct circulating metabolite atlases between NPC patients with different prognoses. We used bootstrap least absolute shrinkage and selection operator (LASSO) on a penalized Cox regression model to select metabolites and constructed a metabolite risk model comprising four microbial metabolites in a training cohort (*n* = 202), and validated it in an independent test cohort (*n* = 201) and an external validation cohort (*n* = 180). The model stratified patients into three risk groups. Patients in the low‐risk group had optimal DFS, DMFS, and OS, compared with those in the intermediate‐risk group. High‐risk patients had poor survival across all clinical endpoints. Furthermore, patients in the intermediate‐risk group could benefit from induction chemotherapy. In addition, we generated a nomogram integrating the risk model, N stage, and plasma EBV‐DNA load, which further enhanced the predictive accuracy of the metabolite risk model. Collectively, we developed and validated a robust predictive model based on serum metabolites, promoting risk stratification and enhancing treatment outcomes in patients with NPC. We identified distinct circulating metabolite atlases between NPC patients with different prognoses in the training cohort (*n* = 202). A risk model, comprising four microbial metabolites, was developed to stratify patients into three risk groups. Patients in the low‐risk group had optimal DFS, DMFS, and OS, compared with those in the intermediate‐risk group. High‐risk patients had poor survival across all clinical endpoints. Findings were validated using an independent test cohort (*n* = 201) and an external validation cohort (*n* = 180). Specifically, a nomogram integrating the risk model, N stage, and plasma EBV‐DNA load enhanced predictive accuracy. Moreover, patients benefited from induction chemotherapy with improved survival in the intermediate‐risk group, but not in the low‐risk and high‐risk groups.

## 1 Background

Nasopharyngeal carcinoma (NPC) is prevalent in South China, Southeast Asia, and North Africa, with over 70% of newly diagnosed cases classified as locoregional advanced NPC (LA‐NPC) [1]. In recent years, substantial advancements in survival rates have been made due to the use of platinum‐based induction chemotherapy (IC) followed by concurrent chemoradiotherapy (CCRT) within the framework of intensity­modulated radiotherapy (IMRT) [2, 3, 4]. About 20% of patients undergoing radical treatment develop recurrence or distant metastasis, leading to treatment failure [2]. Currently, the anatomic‐based tumor‐node‐metastasis (TNM) staging system is widely used for prognostication and treatment guidance [5]. However, variations in survival outcomes among patients receiving similar therapeutic regimens at identical stages highlight the limitation of the current risk stratification method. Although great efforts have been made to identify biomarkers that accurately reflect the biological heterogeneity of tumors, such as Epstein‐Barr virus (EBV)‐DNA, miRNA, and mRNA [6, 7, 8, 9], there is an urgent need to identify novel biomarkers.

Liquid biopsy can reflect the alterations in circulating constituents correlated with cancers in a clinically feasible and minimally invasive manner [10]. In NPC patients, the role of plasmic EBV DNA copy number in the treatment response and prognosis prediction of NPC patients is widely recognized. The EBV DNA level before treatment has been used to predict the patient's prognosis for a long time [11, 12]. Several recent studies have further found that dynamic changes of plasma EBV DNA during treatment can more accurately stratify patients with different prognoses and guide treatment [6, 13]. Moreover, the integration of EBV DNA copy number with tumor stage is also believed to further enhance the predictive ability for prognosis and guide treatment selection [5, 14]. However, the lack of detection standardization in EBV DNA measurement remains a big clinical challenge. The low consistency of detection results among different medical institutions poses a challenge for broader application [15, 16].

Efforts are still being made to search for other biomarkers. The level of circulating cell‐free DNA 5‐hydroxymethylcytosine has been found to stratify NPC with different prognoses [17]. Besides, the plasma proteins, cytokine profile, and circulating immune subset counts, particularly T cells, have also demonstrated their predictive value in the prognosis of NPC patients [18, 19, 20]. In addition, two recent studies have reported that circulating inflammation signatures composed of various blood inflammatory factors or nutritional parameters have promising efficacy in predicting the prognosis of patients and might help guide individualized treatment in both local and metastatic NPC [21, 22].

Metabolic dysregulation is a hallmark of cancer, and metabolites are downstream products of alterations at the genetic and proteomic levels, shaped by endogenous and exogenous factors [23]. Circulating metabolites act as sensitive indicators of pathological variation and hold potential as tumor biomarkers [24, 25]. It is noteworthy that gut microbiota exerts a large effect on host blood metabolites [26]. The gut microbial metabolites can enter the bloodstream and influence the biological behaviors of cancer cells in the distal organs, serving as promising biomarkers for cancer early detection [27, 28, 29, 30, 31]. A recent study reports a plasma microbial metabolite panel for differentiating normal, adenoma, and colorectal cancer [32]. However, the clinical significance of circulating microbial metabolites in cancer prognosis, particularly NPC, remains unexplored.

In this study, we performed a cohort study to depict the pretreatment serum metabolite profiles with the aim of developing and validating a microbial metabolite risk model for distinguishing the likelihood of tumor relapse after radical treatment in patients with LA‐NPC. We further explored the possibility of this risk model for evaluating the efficacy of IC.

## 2 Results

### 2.1 Patient Characteristics

**FIGURE 1:**
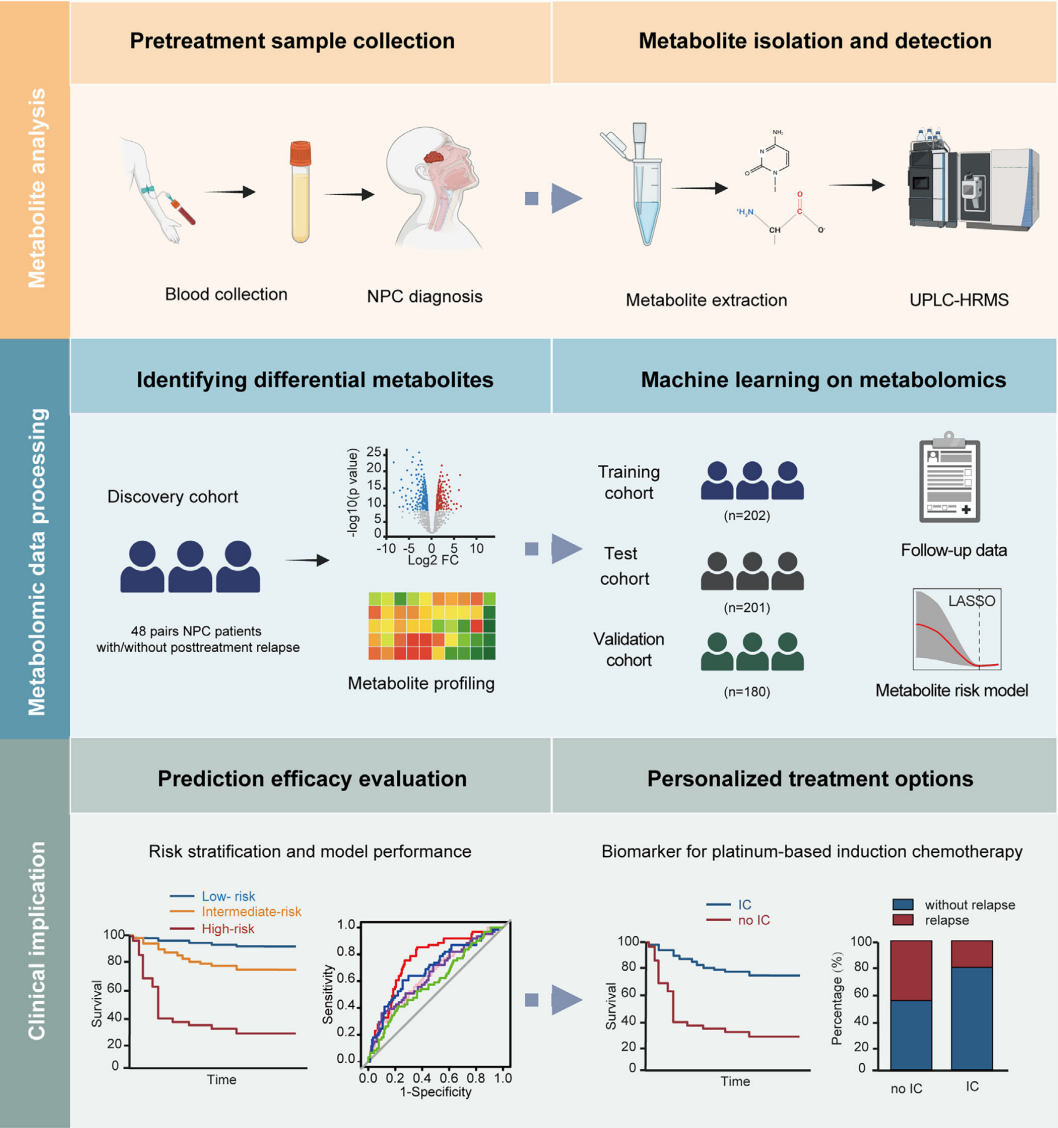
The overall design of our study. IC, induction chemotherapy; LASSO, the least absolute shrinkage and selection operator; NPC, nasopharyngeal carcinoma; UPLC‐HRMS, ultra‐performance liquid chromatography‐high resolution mass spectrometry.

**FIGURE 2:**
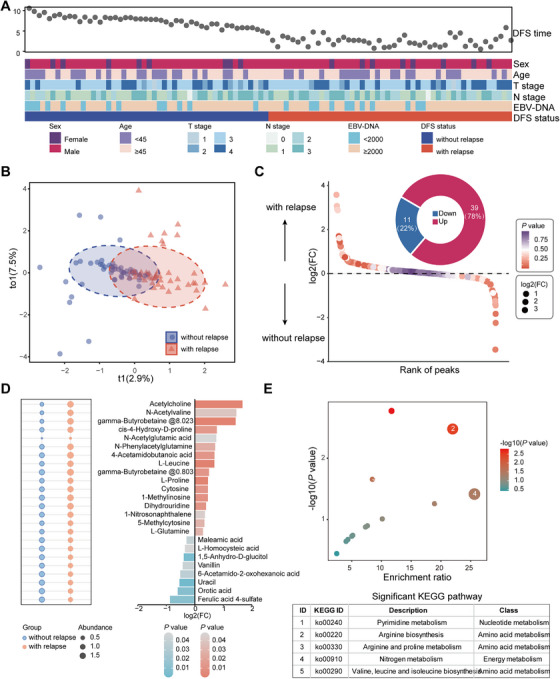
Serum metabolite profile in LA‐NPC. (A) Clinical characteristics of 48 paired LA‐NPC patients who experienced with (*n* = 48, red) or without (*n* = 48, blue) tumor relapse in the discovery cohort. (B) OPLS‐DA plot exhibits a global metabolic difference between patients who experienced with or without tumor relapse. (C) Waterfall plot and donut chart exhibit the number and percentage of up‐regulated and down‐regulated metabolites between patients who experienced with or without tumor relapse. (D) Normalized abundance and log2(FC) of differential metabolites. (E) Metabolite pathway enrichment analysis of differential metabolites. FC, fold change; KEGG, Kyoto Encyclopedia of Genes and Genomes; LA‐NPC, locoregional advanced nasopharyngeal carcinoma; OPLS‐DA, orthogonal partial least squares‐discriminant analysis.

We enrolled a total of 679 non‐metastatic LA‐NPC patients (mean [SD] age, 45.3 [10.8] years; 505 [74.4%] male) in this study (Figure 1, Figure S1). A subset of 96 patients was recruited as a discovery cohort, including 48 paired patients with or without posttreatment tumor relapse, who were strictly matched by age, sex, T stage, N stage, TNM stage, and pretreatment plasma EBV‐DNA load (Figure 2A, Table S1). The time of disease‐free survival (DFS) was significantly shorter in patients experiencing tumor relapse compared to those without tumor relapse (Figure S2A). Patient characteristics of the training, test, and validation cohorts are listed in Table 1. The platinum‐based induction chemotherapy was accepted by 82 (40.6%), 100 (49.8%), and 165 (91.7%) patients in the training, test, and validation cohorts, with a median follow‐up (interquartile range, IQR) of 70.2 (54.2–96.6), 82.1 (55.7–97.3), and 85.6 (59.6–94.0) months, respectively.

**TABLE 1:** Clinical characteristics of patients in the training, test, and validation cohort.

	Training cohort (*n* = 202)				Test cohort (*n* = 201)				Validation cohort (*n* = 180)			
	No. of Patients	Low‐risk (%)	Intermediate‐risk (%)	High‐risk (%)	No. of Patients	Low‐risk (%)	Intermediate‐risk (%)	High‐risk (%)	No. of Patients	Low‐risk (%)	Intermediate‐risk (%)	High‐risk (%)
**Age/years**												
<45	119	65 (67.0)	43 (51.2)	11 (52.4)	111	67 (57.3)	38 (55.1)	6 (40.0)	50	28 (30.4)	21 (27.3)	1 (9.1)
≥45	83	32 (33.0)	41 (48.8)	10 (47.6)	90	50 (42.7)	31 (44.9)	9 (60.0)	130	64 (69.6)	56 (72.7)	10 (90.9)
**Sex**												
Female	52	29 (29.9)	17 (20.2)	6 (28.6)	45	31 (26.5)	12 (17.4)	2 (13.3)	60	33 (35.9)	23 (29.9)	4 (36.4)
Male	150	68 (70.1)	67 (79.8)	15 (71.4)	156	86 (73.5)	57 (82.6)	13 (86.7)	120	59 (64.1)	54 (70.1)	7 (63.6)
**WHO pathological type**												
Undifferentiated non‐keratinising	195	95 (97.9)	81 (96.4)	19 (90.5)	198	115 (98.3)	69 (100)	14 (6.7)	179	92 (100)	76 (98.7)	11 (100)
Other pathological types	7	2 (2.1)	3 (3.6)	2 (9.5)	3	2 (1.7)	0 (0.0)	1 (93.3)	1	0 (0.0)	1 (1.3)	0 (0.0)
**T stage**												
T1	3	1 (1.0)	2 (2.4)	0 (0.0)	11	6 (5.1)	5 (7.2)	0 (0.0)	2	0 (0.0)	2 (2.6)	0 (0.0)
T2	17	11 (11.3)	5 (5.9)	1 (4.8)	15	8 (6.8)	7 (10.1)	0 (0.0)	22	12 (13.0)	9 (11.7)	1 (9.1)
T3	123	61 (62.9)	53 (63.1)	9 (42.9)	123	77 (65.8)	36 (52.2)	10 (66.7)	103	56 (60.9)	42 (54.5)	5 (45.5)
T4	59	24 (24.7)	24 (28.6)	11 (52.4)	52	26 (22.2)	21 (30.4)	5 (33.3)	53	24 (26.1)	24 (31.2)	5 (45.5)
**N stage**												
N0	6	3 (3.1)	2 (2.4)	1 (4.8)	12	8 (6.8)	4 (5.8)	0 (0.0)	3	3 (3.3)	0 (0.0)	0 (0.0)
N1	110	54 (55.7)	46 (54.8)	10 (47.6)	94	65 (55.6)	24 (34.8)	5 (33.3)	28	14 (15.2)	13 (16.9)	1 (9.1)
N2	53	28 (28.9)	21 (25.0)	4 (19.0)	71	34 (29.1)	29 (42.0)	8 (53.3)	122	64 (69.6)	52 (67.5)	6 (54.5)
N3	33	12 (12.4)	15 (17.9)	6 (28.6)	24	10 (8.5)	12 (17.4)	2 (13.3)	27	11 (12.0)	12 (15.6)	4 (36.4)
**TNM stage**												
III	117	63 (64.9)	48 (57.1)	6 (28.6)	126	81 (69.2)	37 (53.6)	8 (53.3)	101	58 (63.0)	40 (51.9)	3 (27.3)
IV	85	34 (35.1)	36 (42.9)	15 (71.4)	75	36 (30.8)	32 (46.4)	7 (46.7)	79	34 (37.0)	37 (48.1)	8 (72.7)
**EBV‐DNA (copies/mL)**												
<2000	85	40 (41.2)	34 (40.5)	11 (52.4)	85	57 (48.7)	25 (36.2)	3 (20.0)	NA	NA	NA	NA
≥2000	117	57 (58.8)	50 (59.5)	10 (47.6)	116	60 (51.3)	44 (63.8)	12 (80.0)	NA	NA	NA	NA
**Induction chemotherapy**												
no	120	48 (49.5)	55 (65.5)	17 (81.0)	101	59 (50.4)	33 (47.8)	9 (60.0)	15	9 (9.8)	5 (6.5)	1 (9.1)
yes	82	49 (50.5)	29 (34.5)	4 (19.0)	100	58 (49.6)	36 (52.2)	6 (40.0)	165	83 (90.2)	72 (93.5)	10 (90.9)
**Disease progression**												
no	141	88 (90.7)	46 (54.8)	7 (33.3)	150	104 (88.9)	41 (59.4)	5 (33.3)	128	83 (90.2)	43 (55.8)	2 (18.2)
yes	61	9 (9.3)	38 (45.2)	14 (66.7)	51	13 (11.1)	28 (40.6)	10 (66.7)	52	9 (9.8)	34 (44.2)	9 (81.8)
**Distant metastasis**												
no	169	91 (93.8)	67 (79.8)	11 (52.4)	167	107 (91.5)	52 (75.4)	8 (53.3)	147	87 (94.6)	55 (71.4)	5 (45.5)
yes	33	6 (6.2)	17 (20.2)	10 (47.6)	34	10 (8.5)	17 (24.6)	7 (46.7)	33	5 (5.4)	22 (28.6)	6 (54.5)
**Death**												
no	154	89 (91.8)	58 (69.0)	7 (33.3)	167	111 (94.9)	49 (71.0)	7 (46.7)	162	89 (96.7)	66 (85.7)	7 (63.6)
yes	48	8 (8.2)	26 (31.0)	14 (66.7)	34	6 (5.1)	20 (29.0)	8 (53.3)	18	3 (3.3)	11 (14.3)	4 (36.4)

Abbreviations: EBV, Epstein‐Barr virus; TNM, tumor‐node‐metastasis; WHO, World Health Organization.

### 2.2 Alteration of Serum Metabolite Profile

We first compared the serum metabolite profiles using untargeted metabolomic analysis in 48 paired LA‐NPC patients with or without posttreatment tumor relapse. Principal component analysis (PCA) showed that the quality control (QC) samples were tightly clustered in the middle of the PCA score plot, corroborating the performance stability of this study (Figure S2B). As demonstrated by orthogonal partial least squares‐discriminant analysis (OPLS‐DA), we observed a clear distinction in serum metabolites between NPC patients who experienced relapse and those who did not (Figure 2B). We found 50 significantly differential features in the comparison of patients with tumor relapse with those without (*p *< 0.05; absolute value of Log2[Fold Change] > 0.263, Figure 2C). Peaks were further annotated, and 24 endogenous metabolites were identified (Figure 2D), among which 16 metabolites were enriched in NPC patients with tumor relapse, and 8 were diminished (Figure S3, Table S2). Importantly, most of the differential metabolites (18 out of 24, 75%) were classified as gut microbial metabolites according to a publicly available dataset [33]. The differential metabolites covered a diverse spectrum of biochemicals, including amino acids (38%), pyrimidines (17%), fatty acids (13%), and so on (Figure S4). Pathway analysis showed that several metabolic pathways, including pyrimidine metabolism, arginine metabolism, and arginine and proline metabolism, were disturbed in patients with tumor relapse (Figure 2E). Our data imply that the malignant progression of NPC is intensely related to metabolic dysregulation.

### 2.3 Construction of a Serum Microbial Metabolite Risk Model

**FIGURE 3:**
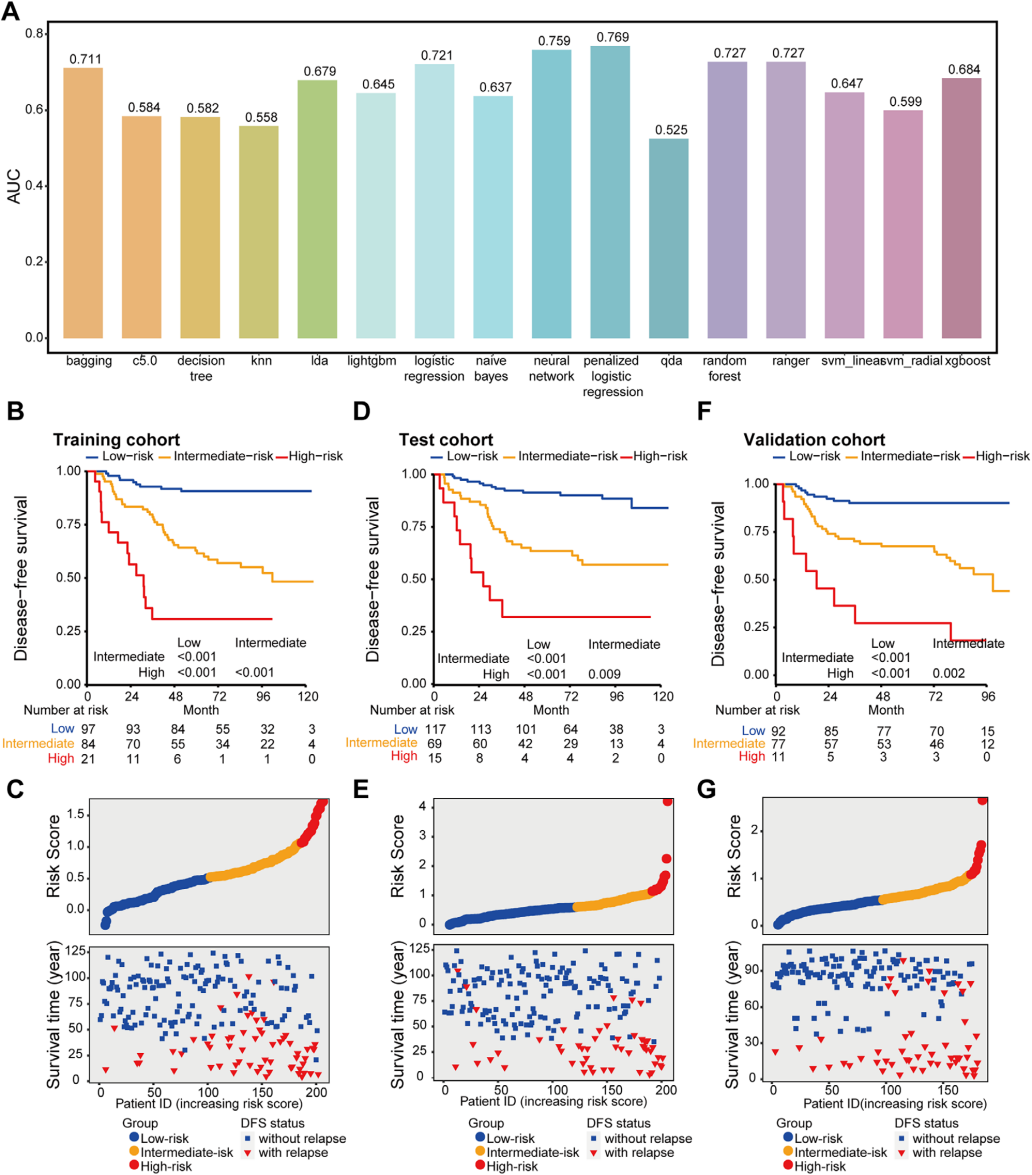
Stratification of LA‐NPC patients according to the microbial metabolite risk model. (A) Comparison of multiple machine learning models with area under the curve (AUC) as performance metric. (B) Kaplan–Meier curves of disease‐free survival in the training cohort (*n* = 202) according to the microbial metabolite risk model. We calculated the *p* values with the unadjusted log‐rank test. (C) Risk score distribution with survival status and time in the training cohort. Red, orange, and blue dots represent high‐risk, intermediate‐risk, and low‐risk scores, respectively. Red triangles mean relapse cases, and blue squares mean no relapse during observation. (D) Kaplan–Meier curves of disease‐free survival in the test cohort (C; *n* = 201) according to the microbial metabolite risk model. We calculated the *p* values with the unadjusted log‐rank test. (E) Risk score distribution with survival status and time in the test cohort. Red, orange, and blue dots represent high‐risk, intermediate‐risk, and low‐risk scores, respectively. Red triangles mean relapse cases, and blue squares mean no relapse during observation. (F) Kaplan–Meier curves of disease‐free survival in the validation cohort (*n* = 180) according to the microbial metabolite risk model. We calculated the *p* values with unadjusted log‐rank test. (G) Risk score distribution with survival status and time in validation cohort. Red, orange, and blue dots represent high‐risk, intermediate‐risk, and low‐risk scores, respectively. Red triangles mean relapse cases, and blue squares mean no relapse during observation.

To develop a serum metabolite‐based risk model for predicting tumor relapse, we employed a comprehensive suite of 16 distinct machine learning algorithms in the training cohort. Among these algorithms, penalized regression exhibited the highest area under the curve (AUC) value for distinguishing patients with tumor relapse from those without (Figure 3A). Consequently, we employed the least absolute shrinkage and selection operator (LASSO), a form of penalized regression, and ultimately identified four key metabolites that were closely associated with DFS (Figure S5A,B, Table S3). The analysis of metabolite abundance revealed that these four metabolites exhibited significant differences between patients with distinct clinical outcomes. Specifically, three metabolites (4‐Acetamidobutanoic acid, L‐Proline, and N‐Phenylacetylglutamine) were found to be elevated in patients experiencing tumor relapse, whereas the Vanillin level was reduced in this group (Figure S5C).

To assess the disease risk for dependent patients, we generated a risk score for each patient with the following formula: risk score = (0.176 × abundance of 4‐Acetamidobutanoic acid) + (0.185 × abundance of L‐Proline) + (0.257 × abundance of N‐Phenylacetylglutamine) − (0.107 × abundance of Vanillin). Receiver operating characteristic (ROC) analysis showed that the microbial metabolite risk model performed well in predicting DFS, distant metastasis‐free survival (DMFS), and overall survival (OS), with the highest AUC values (DFS, 0.761, 95%CI 0.690–0.832; DMFS, 0.724, 95%CI 0.624–0.825; OS, 0.771, 95%CI 0.694–0.849), compared with every single metabolite (all *p *< 0.05, Figure S5D).

Next, we applied X‐tile plots to determine the optimal cutoff values (0.47 and 1.01) for classifying patients into three risk groups (Figure S6A–C). Particularly, the three up‐regulated metabolites in patients experiencing tumor relapse exhibited a continuous increasing pattern along with the various risk groups (Figure S6D). This assay allotted 97 out of 202 patients (48.0%) to the low‐risk group, 84 (41.6%) to the intermediate‐risk group, and 21 (10.4%) to the high‐risk group. Survival outcomes varied markedly among the three risk groups. Notably, NPC patients classified into the low‐risk group had optimal DFS (HR 5.64, 95%CI 2.73–11.68, *p *< 0.001), DMFS (HR 3.53, 95%CI 1.39–8.95, *p* = 0.004), and OS (HR 4.02, 95%CI 1.82–8.89, *p *< 0.001), compared with patients in the intermediate‐risk group. On this note, we observed that patients in the high‐risk group had the worst survivals across all clinical endpoints (Figure 3B, Figure S7A,B). We further found that patients with low‐risk scores demonstrated a significantly reduced incidence of tumor relapse compared to those with intermediate‐ and high‐risk scores (Figure 3C).

### 2.4 Validation of the Serum Microbial Metabolite Risk Model

We then validated the stability of the serum microbial metabolite risk model in the internal test cohort. The risk model classified 117 patients (58.2%) into the low‐risk group, 69 (34.3%) into the intermediate‐risk group, and 15 (7.5%) into the high‐risk group, using the same cutoff developed in the training cohort. Compared to patients in the low‐risk group, patients in the high‐risk group had inferior DFS (HR 11.08, 95%CI 4.83–25.42, *p *< 0.001), DMFS (HR 8.70, 95%CI 3.29–23.00, *p *< 0.001), and OS (HR 17.77, 95%CI 6.13–51.51, *p *< 0.001). Intermediate‐risk patients also had poorer survivals than low‐risk patients (Figure 3D,E, Figure S7C,D).

To further substantiate that the serum microbial metabolite risk model had similar prognostic value in different populations, we applied it to the external validation cohort. Patients were classified into three groups: low‐risk group (*n* = 92, 51.1%), intermediate‐risk (*n* = 77, 42.8%), and high‐risk (*n* = 11, 6.1%). Kalam–Meier curves demonstrated that patients in these three risk strata exhibited markedly distinct survival outcomes (all *p *< 0.05, Figure 3F,G, Figure S7E,F).

**FIGURE 4:**
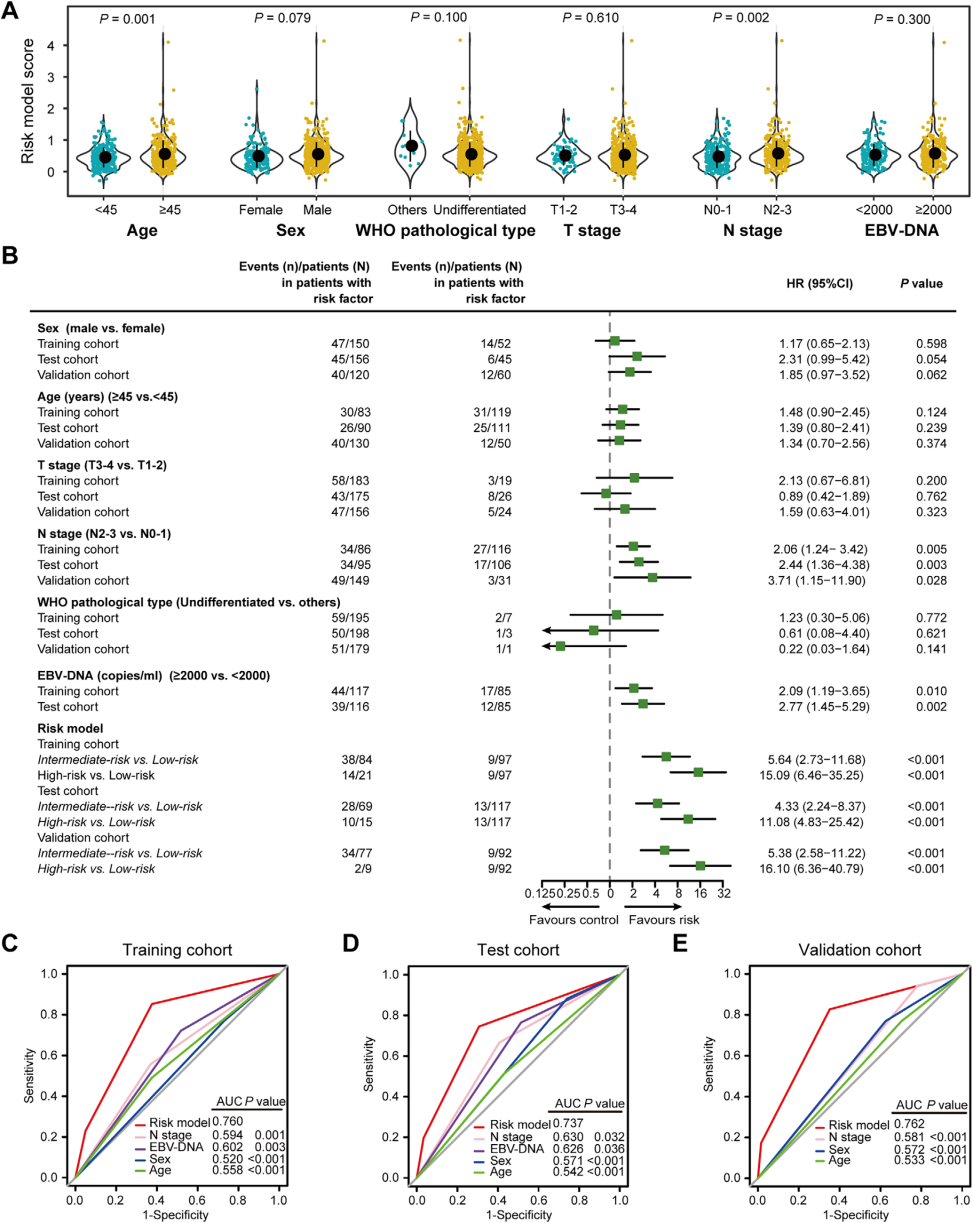
Performance of the microbial metabolite risk model and clinical characteristics. (A) Violin plots show the risk model score distribution by clinical phenotype. We calculated the *p* values with Student *t*‐test. (B) Univariate Cox regression analysis of the microbial metabolite risk model and clinical characteristics with DFS. (C)–(E) ROC analysis compared the risk model with clinical characteristics for DFS in the training cohort (C), test cohort (D), and validation cohort (E). EBV, Epstein‐Barr virus; HR, hazard ratio; ROC, receiver operating characteristic curve.

Previous studies have consistently shown that N‐Phenylacetylglutamine exhibits the strongest correlation with the age‐related gut microbiome [34]. We subsequently investigated the associations between the metabolite risk model and various clinical variables. Our analysis revealed that both advanced age and higher N stage were significantly associated with elevated risk model scores (Figure 4A, Figure S8A). Univariate Cox regression analysis indicated that the metabolite risk model, N stage, and plasma EBV‐DNA load were significantly associated with DFS across all three cohorts (Figure 4B). After multivariate adjustment by clinical variables, the risk model remained an independent prognostic factor in all three cohorts (Tables S4–S6).

ROC analysis demonstrated that the metabolite risk model outperformed both the N stage and plasma EBV‐DNA load in predicting DFS in all three cohorts (all *p *< 0.05, Figure 4C–E), which was further corroborated by the time‐dependent AUC analysis (Figure S8B–D). The calibration curve and decision curve analysis also confirmed the excellent performance of the metabolite risk model (Figure S9). We then conducted subgroup analysis based on clinical covariates associated with tumor relapse in the combined training and test cohorts. The serum metabolite risk model consistently maintained outstanding performance in distinguishing clinical outcomes in patients stratified by N stage (all *p *< 0.05, Figure S10), as well as by plasma EBV‐DNA load (all *p *< 0.05, Figure S11).

### 2.5 Building a Predictive Nomogram

**FIGURE 5:**
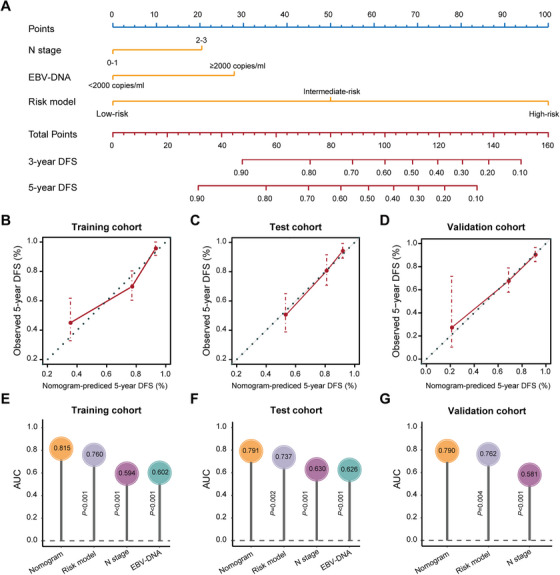
Nomogram to predict the risk of DFS in LA‐NPC. (A) Nomogram to predict DFS. (B)–(D) Calibration curves of the nomogram to predict DFS at 5 years in the training cohort (B), test cohort (C), and validation cohort (D). The actual DFS is plotted on the *y*‐axis, and nomogram predicted probability is plotted on the x‐axis.

To provide a clinically applicable method that predicts an individual's probability of tumor relapse, we used a nomogram to build a predictive model through integrating different risk factors. Based on the multivariate analysis of DFS (Table S4), we generated a nomogram to predict DFS in the training cohort (Figure 5A). This nomogram integrates the metabolite risk model, N stage, and plasma EBV‐DNA load, among which the metabolite risk model had the highest concordance index (C‐index, Table S7). The calibration plots of the nomogram for 5‐year DFS were predicted well in the training cohort (C‐index 0.774, 95%CI 0.720–0.828), test cohort (C‐index 0.764, 95%CI 0.701–0.828), and validation cohort (C‐index 0.731, 95%CI 0.668–0.795; Figure 5B–D). ROC analysis showed that DFS status was more accurately predicted by the nomogram than by other risk factors across all cohorts (*p *< 0.05, Figure 5E–G).

### 2.6 Treatment Stratification Based on the Metabolite Risk Model

**FIGURE 6:**
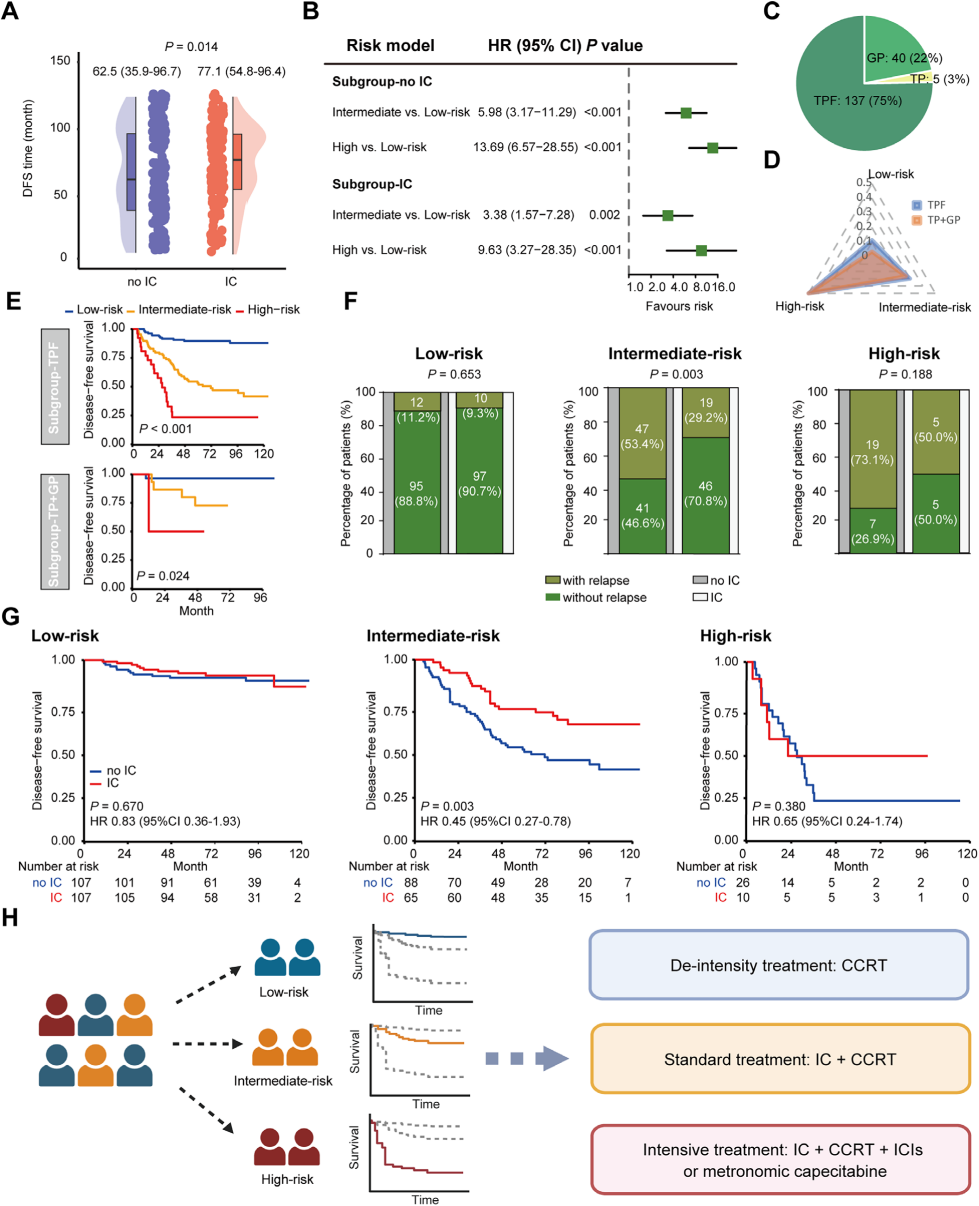
Treatment stratification based on the microbial metabolite risk model. (A) The survival time of LA‐NPC patients receiving either IC or no IC. We calculated the *p* values with Student *t*‐test. (B) Forest plot summarizing HRs with 95%CI for the risk model split by IC subgroups in the combined training and test cohorts. (C) Percentage of LA‐NPC patients receiving specific regimens of IC. (D) Risk model score distribution of LA‐NPC patients receiving specific IC regimens. (E) Kaplan–Meier curves of DFS in the IC and no IC subgroups according to the microbial metabolite risk model. (F) Association of the relapse status with IC stratified by the risk model. We calculated *p* values by *χ* ^2^ test or Fisher's exact test. (G) Treatment stratification of DFS in patients with the low‐risk scores (left panel), intermediate‐risk scores (median panel) and high‐risk score (right panel). We calculated *p* values by unadjusted long‐rank test and HRs using a univariate Cox regression analysis. (H) Proposed risk‐adapted personalized treatment strategies based on the microbial metabolite risk model. CCRT, concurrent chemoradiotherapy; CI, confidence interval; HRs, hazard ratios; IC, induction chemotherapy; ICIs, immune checkpoint inhibitors.

We then explored whether the metabolite risk model could assist clinical therapeutic decision‐making. In the combined training and test cohorts, 182 (45.2%) patients were treated with IC plus CCRT, while the others received CCRT alone. Patients who received IC had comparably favorable survival outcomes relative to those who did not receive IC (Figure 6A). Univariate Cox regression analysis confirmed the prognostic value of the metabolite risk model in both IC and no IC subgroups (Figure 6B). In the IC subgroup, 75% of patients were administered with 5‐fluorouracil, docetaxel, and cisplatin (TPF) regimens, whereas the remaining 25% received either gemcitabine plus cisplatin (GP) or docetaxel plus cisplatin (TP) regimens (Figure 6C). The distribution of risk model scores among these groups was comparable (Figure 6D). After stratification by IC regimens, the risk model retained excellent predictive power for clinical outcomes (all *p *< 0.05, Figure 6E). We subsequently compared the efficacy of IC across different risk groups. In the low‐risk group, there were no obvious differences in tumor relapse and survival outcomes between patients who received IC and those who did not. Patients who received IC in the intermediate‐risk group had better DFS (HR 0.45, 95%CI: 0.27–0.78; *p* = 0.003), DMFS (HR 0.35, 95%CI: 0.16–0.78; *p* = 0.007), and OS (HR 0.41, 95%CI: 0.21–0.79; *p* = 0.006) than those who did not. For high‐risk patients, IC tended to improve survival but had no statistical significance (Figures 6F–G, Figure S12). Furthermore, the clinical characteristics between patients with IC or without IC are comparable (Table S8). And the Cox multivariate analysis proved that receiving induction chemotherapy is an independent prognostic factor for better disease‐free survival, distant‐metastasis‐free survival, and overall survival in patients with intermediate‐risk (Table S9), further supporting that patients in intermediate‐risk group could benefit from induction chemotherapy.

These findings prompt us to propose a risk‐stratified treatment strategy based on the metabolite risk model for LA‐NPC patients (Figure 6H). Specifically, patients in the low‐risk group have favorable survival and should accept CCRT alone. For those in the intermediate‐risk group who are prone to benefit from IC, IC followed by CCRT remains prioritized. High‐risk patients, who face poor prognoses, require a more intensive treatment approach.

## 3 Discussion

In this study, we conducted a multi‐center cohort study with a large sample size to capture the pre‐treatment serum metabolic profile of LA‐NPC patients. We generated and validated a microbial metabolite risk model for distinguishing the likelihood of posttreatment tumor relapse. A nomogram integrating the metabolite risk model, N stage, and plasma EBV‐DNA load improved prognostic assessment. Moreover, our model had promise for evaluating the chemotherapy efficacy, potentially facilitating risk‐adapted personalized management for NPC patients.

Prognostic assessment is critical for treatment decisions for cancer patients. Significant efforts have been dedicated to identifying novel prognostic biomarkers in NPC [7, 8, 9, 35]. However, the most widely utilized prognostic tools for NPC remain the TNM staging system and plasma EBV‐DNA load. The integration of the TNM staging system with plasma EBV‐DNA load enhances the accuracy for prognostic assessment in NPC patients; however, it still yields a relatively low accuracy rate below 70% [36]. It is widely recognized that the TNM staging system and plasma EBV‐DNA load primarily assess disease extent by reflecting anatomic location and tumor burden. Both factors predominantly focus on the tumor itself, neglecting the overall physiological state of the human body. The circulating metabolites derived from gut microbiota serve as critical intermediaries linking the gut microbiome to cancer progression, primarily through regulating oncogenic signaling pathways and remodeling the tumor microenvironment [37, 38]. Here, we introduced a risk model consisting of four serum microbial metabolites for assessing prognosis in LA‐NPC. The serum metabolite risk model outperformed the N stage and plasma EBV‐DNA load. More importantly, we developed a nomogram integrating the metabolite risk model, N stage, and plasma EBV‐DNA load, which comprehensively reflects both the tumor features and systemic conditions. This nomogram represents the most accurate tool for prognostic evaluation in patients with LA‐NPC.

Currently, IC followed by CCRT has been established as a standard treatment strategy for LA‐NPC patients. However, evidence supporting its personalized application remains insufficient [4, 39]. Oncologists still wrestle with the challenges of selecting the optimal therapeutic regimens for individuals through balancing the unwanted adverse effects and the achievable survival benefits. In our present study, we stratified NPC patients into three distinct risk groups based on the microbial metabolite risk model. Patients classified as having intermediate‐risk scores could derive significant benefits from the NCCN guideline‐recommended IC regimens, such as TPF, GP, and TP, whereas those categorized as low or high‐risk did not exhibit similar advantages. Based on these findings, we advocate for prioritizing IC followed by CCRT for patients with intermediate‐risk scores. The administration of IC might not bring extra benefits to patients with low‐risk scores, since these patients have excellent survival outcomes. Therefore, this subgroup is the biggest beneficiary of the concept of “de‐intensity treatment and reducing toxicity”. Conversely, for high‐risk patients who were expected to exhibit a worse prognosis, probably due to the adverse effect of risk metabolites, the current treatments did not meet the clinical needs. An intensive treatment strategy employing agents with diverse mechanisms of action, such as the combination of immune checkpoint inhibitors (ICIs) before, during, or after radiotherapy, alongside metronomic capecitabine in adjuvant therapy, may improve the chance of achieving effective disease control.

Intriguingly, all metabolites included in the risk model are relevant to gut microbiota, providing further evidence for the gut‐metabolite‐distal organ axis in cancer pathogenesis. Previous studies have shown that the enrichment of gut microbial metabolites plays a pivotal role in the initiation and progression of cancer [40]. It should be noted that the microbial metabolites featured in the risk model are closely linked to tumors, such as L‐Proline [41, 42, 43]. N‐Phenylacetylglutamine, converted from the dietary phenylalanine by anaerobic microorganisms, might be linked to an increased likelihood of developing incident lethal prostate cancer [44]. Regarding Vanillin, it appears to possess potential for suppressing cancer metastasis by inhibiting PI3K [45]. Although the underlying mechanisms of these metabolites require further investigation, chemoresistance induced by metabolites is likely a remarkable contributor to poor prognosis in high‐risk patients. A comprehensive understanding of microbiota‐derived metabolites could facilitate the exploration and development of innovative therapeutic approaches for cancer, including probiotics, dietary modifications, and fecal microbiota transplantation [46, 47]. Indeed, incorporating microbial metabolites into antitumor therapies can enhance the treatment efficacy [48].

Despite the exciting performance of our metabolite risk model in prognosis prediction in NPC patients, there are some limitations that need to be addressed. Firstly, in this study, we only conducted metabolite detection on the samples collected before the treatment. A serial measurement of these metabolites may be more capable of predicting relapse events or detecting residual disease in NPC patients, but it warrants further investigation. Besides, whether it can truly assist in clinical treatment decisions remains to be verified in the future, as the current exploration and validation are limited to single‐center cohorts. The prospective, large‐scale, multicenter studies are warranted to validate our findings before their clinical application.

## 4 Conclusions

To our knowledge, this cohort study, with the largest sample size to date, offers the most detailed characterization of serum metabolite profiles in LA‐NPC patients with distinct prognoses. The metabolite risk model serves as an effective tool for identifying beneficiaries of IC, thereby guiding therapeutic decisions and prognosis evaluation.

## 5 Materials and Methods

This study was reported according to the Transparent reporting of a multivariable prediction model for individual prognosis or diagnosis statement (TRIPOD, Table S10).

### 5.1 Study Population

We retrospectively collected 679 pre‐treatment serum specimens from LA‐NPC patients with Stage III–IVa disease. Of these, 499 samples were obtained from patients treated at the Sun Yat‐sen University Cancer Center (Guangzhou, China) between July 2010 and November 2016. Among them, 96 serum samples from 48 paired patients, who either experienced post‐treatment tumor relapse within 3 years or remained relapse‐free for over 5 years, were designated as the discovery cohort. In the remaining 403 patients, 102 experienced disease progression, 67 developed distant metastasis, and 82 patients died. Another 180 samples obtained from patients treated at the Affiliated Hospital of Guilin Medical College (Guilin, China) between September 2015 and September 2018 were allocated as an independent validation cohort. In this cohort, 52 patients experienced disease progression, 33 patients developed distant metastasis, and 18 patients died. The clinical characteristics of all patients are presented in Table 1.

None of the patients received any anti‐tumor treatment prior to blood sampling. All magnetic resonance imaging (MRI) scans of the nasopharyngeal and cervical regions were independently evaluated by two radiologists, with any discrepancies resolved through consensus. The TNM stages were reevaluated according to the eighth edition of the American Joint Committee on Cancer (AJCC) staging system. All patients were administered CCRT, with 61.0% receiving an additional 2–3 cycles of IC. The regimens of IC included TPF, GP, and TP, all of which are recommended by the NCCN guidelines. For IMRT, the prescribed doses for the primary gross tumor were 66–72 Gy/ 30–33 fractions, while for the involved nodal regions were 66–70 Gy/30–33 fractions. For CCRT, cisplatin (80–100 mg/m^2^) was administered every 3 weeks for 2–3 cycles during IMRT. The study was conducted in accordance with the REMARK guidelines.

### 5.2 Sample Size Estimation

For sample size estimation for the training cohort, we used the events per variable (EPV) metric [49, 50, 51], a widely accepted method in statistical analyses with the following formula: Sample Size = (Number of Variables × EPV)/(Incidence rate). In our training cohort, the disease progression rate was 0.330 (61/202). We intention to include 4 predictor variables and set the EPV to 10. Accordingly, the required sample size is approximately 133. We thus employed computer‐generated random numbers to classify the 403 NPC patients on average into a training cohort (*n* = 202) and a test cohort (*n* = 201).

### 5.3 Sample Collection and Metabolite Extraction

Fasting peripheral blood samples were collected for serum isolation and subsequently stored at −80°C until use. As we previously described, the metabolites were extracted from serum (50 µL) by the sequential addition of precooled methanol (500 µL), MS‐grade water containing internal standards (150 µL), and chloroform (500 µL). The mixtures were vortexed for 10 min, and centrifuged at 4°C, 15000 rcf for 15 min. Supernatant (300 µL) was transferred to two new tubes (150 µL/tube) for the analysis of positive and negative modes. The extract was evaporated to dryness via a speed vacuum concentrator and stored at −80°C.

### 5.4 Metabolite Profiling Analysis

Untargeted metabolomic analysis was conducted at the ultra‐performance liquid chromatography‐high resolution mass spectrometry (UPLC‐HRMS) platform from the Sun Yat‐sen University Metabolomics Center (Guangzhou, China). Briefly, the metabolites were separated with a Dionex Ultimate 3000 UPLC system with an ACQUITY BEH Hilic column (Waters). The mobile phases employed 95% (A) and 50% acetonitrile (B), both containing 10 mmol/L ammonium acetate. The gradient program was as follows: 0–1 min, 1% B; 1–13 min, 1%–100% B; 13–15 min, 100% B; 15–15.1 min, 100%–1% B; 15.1–18 min, 1% B. The column was sustained at 40°C, with the samples preserved in the autosampler at 10°C. The flow rate was 0.4 mL/min, and the injection volume was 3 µL. The Q‐Exactive Orbitrap mass spectrometer (Thermo Fisher Scientific) with electrospray ionization (ESI) source was performed in full scan mode in combination with ddMS2 monitoring mode for data acquisition. We set up the parameters as follows: spray voltage +3.8 kV/−3.2 kV; capillary temperature 320°C; sheath gas 40 arb; auxiliary gas 10 arb; probe heater temperature 350°C; scan range 70–1050 m/z; resolution 70000. The mass data were captured using Xcalibur 4.1 Software (Thermo Fisher Scientific). For data quality control, the QC sample was prepared by mixing equal volumes of supernatant from each sample.

### 5.5 Metabolomic Data Processing

The raw peak data were extracted using Compound Discovery 3.1 and TraceFinder 4.0 software (Thermo Fisher Scientific). Metabolite features deemed missing were removed based on the 80% rule [52], whereby a peak was retained if it existed in at least 80% of the samples. The peaks with coefficients of variation (CV) below 30% in QC samples were maintained. Undetected values of these peaks were filled with the k‐nearest neighbors (KNN) method using the statTarget package in R [53], yielding a final dataset without missing values. A QC‐based random forest signal correction (QC‐RFSC) approach was employed to remove unwanted signal drift and avoid batch effect. The relative abundance was normalized by comparing each peak area to the median peak area of all detected peaks. Principal component analysis (PCA) was applied to assess data quality in QC samples by the FactoMineR package (v2.11).

### 5.6 Development of a Metabolite Risk Model

The distribution of merged features (positive and negative modes) was depicted by orthogonal partial least squares‐discriminant analysis (OPLS‐DA) with the ropls package (v1.30.0). The features were queried and verified against the mzCloud and ChemSpider databases. We identified differential metabolites using Student *t*‐test in the rstatix package (v0.7.1), and then performed Kyoto Encyclopedia of Genes and Genomes (KEGG) pathway analysis using MetaboAnalyst 6.0. We compared the performance of multiple classification or regression models using the fastml package (v0.3.0). We used bootstrap least absolute shrinkage and selection operator (LASSO) on a penalized Cox regression model to select metabolites for constructing a risk model using the glmnet package (v4.1‐6), and the λ value was chosen with the min (minimum error) criteria under ten‐fold cross‐validation. We calculated a risk score for each patient with a formula derived from the normalized peak areas of metabolites weighted by the coefficients from the Cox model in the training cohort.

### 5.7 Statistical Analysis

The primary endpoint was DFS, and the secondary endpoints included DMFS and OS. DFS was defined as the period from the first date of treatment to the date of relapse at any site or death from any cause, whichever occurred first; DMFS as the period to the date of first distant metastasis; and OS as the period to the date of death from any cause.

We used X‐tile software (v3.6.1) to identify the optimal cutoff for classifying patients into low, median, or high‐risk groups in the training cohort [54]. The *χ* ^2^ test or Fisher's exact test was used to compare categorical variables. We assessed the survival probability by the Kaplan–Meier method and the log‐rank test using the survminer package (v0.4.9). We calculated Hazard ratios (HRs) by univariable Cox analysis and tested independent prognostic factors by multivariable Cox analysis with the backward stepwise method. We utilized coefficients of the Cox model to generate nomograms, integrating the metabolite risk model, N stage, and plasma EBV‐DNA load, using the rms package (v6.5). We assessed calibration curves graphically by comparing the observed rates with the nomogram's predicted probabilities. Concordance index (C‐index) was calculated by the survival package (v3.4). We tested predicted efficiency by Receiver operating characteristic (ROC) curves and time‐dependent AUC analysis by pROC (v1.18.0) and survivalROC (v1.0.3.1). All statistical tests were performed in SPSS (v22.0) and R (v4.2.2) with two‐sided tests, and a *p* value < 0.05 was considered significant.

## Author Contributions

Na Liu, Yu‐Min Hu, and Ying‐Qing Li had full access to all the data in the study and take responsibility for the integrity of the data and the accuracy of the data analysis. Study Concept and design: Na Liu, Yu‐Min Hu, and Ying‐Qing Li. Acquisition, analysis, or interpretation of data: Jun‐Yan Li, Yao Yao, Xi‐Rong Tan, Nan Si, Wei Jiang, Ying‐Qi Lu, Jia‐Hao Dai, Tian‐Tian Yu, Hao‐Cheng Hu, Yu‐Fei Duan, Sen‐Yu Feng, Sai‐Wei Huang, Ye‐Lin Liang, Sha Gong, Na Liu, Yu‐Min Hu, Ying‐Qing Li. Statistical analysis: Na Liu, Jun‐Yan Li, Xi‐Rong Tan, and Yao Yao. Drafting of the manuscript: Jun‐Yan Li, Xi‐Rong Tan, and Na Liu. Critical revision of the manuscript for important intellectual content: Jun‐Yan Li, Yao Yao, Xi‐Rong Tan, Na Liu, Yu‐Min Hu, and Ying‐Qing Li. Obtained funding: Jun‐Yan Li and Na Liu. Administrative, technical, or material support: Na Liu, Yu‐Min Hu, and Ying‐Qing Li. Study supervision: Na Liu, Yu‐Min Hu, and Ying‐Qing Li. All authors have read and approved the final manuscript.

## Funding

This study was supported by grants from the National Natural Science Foundation of China (82403970 to Dr Li, 82172592, 82461160321, 82172806 to Dr Liu), the Key Research and Development Program of Guangzhou City (202206010013 to Dr Liu), the Guangdong Basic and Applied Basic Research Foundation (2022A1515220010 to Dr Liu), the Young Talents Program of Sun Yat‐sen University Cancer Center (YTP‐SYSUCC‐0010 to Dr Liu), the Key Research and Development Program of Guangxi (AB25069454 to Dr. Jiang), and the Science Research and Technology Development Program of Guilin (20230127‐1 to Dr. Jiang).

## Ethics Statement

This retrospective analysis of anonymous data received approval from the institutional ethical review boards of both hospitals, and all patients signed written informed consent (B2021‐311).

## Conflicts of Interest

The authors declare no conflicts of interest.

## Supporting information




**Supporting Table 1**: Clinical characteristics of 48 paired patients with LA‐NPC experienced with or without tumor relapse in the discovery cohort.
**Supporting Table 2**: Annotated differential metabolites in serum of 48 paired NPC patients with or without relapse in the discovery cohort.
**Supporting Table 3**: A model of 4 metabolites with prognostic implication in the training cohort.
**Supporting Table 4**: Multivariable Cox regression analysis of prognostic factors of patients in the training cohort.
**Supporting Table 5**: Multivariable Cox regression analysis of prognostic factors of patients in the test cohort.
**Supporting Table 6**: Multivariable Cox regression analysis of prognostic factors of patients in the validation cohort.
**Supporting Table 7**: The C‐index of the nomogram and risk factors for prediction of disease‐free survival (DFS) in the training, test, and validation cohorts.
**Supporting Table 8**: Clinical characteristics of patients with LA‐NPC in intermediate‐risk group experienced with or without IC.
**Supporting Table 9**: Multivariable Cox regression analysis of prognostic factors of patients in the intermediate‐risk group.
**Supporting Table 10**: Transparent reporting of a multivariable prediction model for individual prognosis or diagnosis statement (TRIPOD)
**Supporting Figure 1**: Study population. Abbreviations: LA‐NPC, locoregional advanced nasopharyngeal carcinoma; TNM, tumor‐mode‐metastasis; LASSO, the least absolute shrinkage and selection operator.
**Supporting Figure 2**: Characteristics of LA‐NPC patients in the discovery cohort. **(A)** Survival time of patients experienced with (*n* = 48) or without (n = 48) tumor relapse. We calculated the *p* values with Student t‐test. **(B)** Principal component analysis (PCA) plot showed the distribution of QC and samples.
**Supporting Figure 3**: Differential serum metabolites in LA‐NPC patients with or without tumor relapse in the discovery cohort. The comparisons were conducted with Student t‐test. The center line represents the median normalized abundance, and the box bound represents the inter‐quartile range.
**Supporting Figure 4**: The category percentage of differential metabolites. **(A)** The categories of the total differential metabolites, **(B)** enriched metabolites and **(C)** diminished metabolites in LA‐NPC patients with or without tumor relapse.
**Supporting Figure 5**: Construction of the microbial metabolite risk model in the training cohort. **(A)** LASSO coefficient profiles of the candidate metabolites for model construction. **(B)** Ten‐time cross‐validations in LASSO were performed to tune the parameter selection. Dotted vertical lines are drawn at the optimal values by minimum criteria. **(C)** Left panel: Comparison of the normalized abundance of four metabolites identified by LASSO. We calculated the *p* values with Student t‐test. Right panel: Forest plot of the four metabolites by univariate Cox regression analysis. **(D)** ROC analysis compared the risk model with each single risk metabolite for disease‐free survival (DFS), distant metastasis‐free survival (DMFS), and overall survival (OS).
**Supporting Figure 6**: Determination of optimum cutoff points for microbial metabolite risk model. **(A‐C)** Thresholds selection using X‐tile in the training cohort. **(D)** Bar plots showed the change trends of four risk metabolites determined by the risk model in the training cohort (left), test cohort (median) and validation cohort (right).
**Supporting Figure 7**: Kaplan‐Meier curves of DMFS and OS in the training, test and validation cohorts according to the microbial metabolite risk model. **(A‐B)** Kaplan‐Meier curves of DMFS (A) and OS (B) in the training cohort by the risk model. **(C‐D)** Kaplan‐Meier curves of DMFS (C) and OS (D) in the test cohort by the risk model. **(E‐F)** Kaplan‐Meier curves of DMFS (E) and OS (F) in the validation cohort by the risk model. We calculated the *p* values with unadjusted log‐rank test.
**Supporting Figure 8**: Prognostic performance of the microbial metabolite risk model and clinical characteristics. **(A)** Boxplots represented the risk model score determined by N stage. **(B‐D)** Time‐dependent AUC analysis compared the microbial metabolite risk model with clinical characteristics for DFS in a continuous period in the training cohort (B), test cohort (C), and validation cohort (D).
**Supporting Figure 9**: Calibration curve (**A‐C**) and decision curve analysis (**D‐E**) of the metabolite risk model in training, test, and validation cohorts.
**Supporting Figure 10**: Kaplan‐Meier curves of DFS, DMFS and OS according to the microbial metabolite risk model stratified by N stage. **(A‐C)** DFS (A), DMFS (B) and OS (C) in the stage N0‐1 group according to the microbial metabolite risk model. **(D‐E)** DFS (D), DMFS (E) and OS (F) in the stage N2‐3 group according to the microbial metabolite risk model. We calculated the *p* values with unadjusted long‐rank test.
**Supporting Figure 11**: Kaplan‐Meier curves of DFS, DMFS and OS according to the microbial metabolite risk model stratified by plasma EBV‐DNA load. **(A‐C)** DFS (A), DMFS (B) and OS (C) in the low plasma EBV‐DNA load group (<2000 copies/ml) according to the microbial metabolite risk model. **(D‐E)** DFS (D), DMFS (E) and OS (F) in the high plasma EBV‐DNA load group (≥2000 copies/ml) according to the microbial metabolite risk model. We calculated the *p* values with unadjusted long‐rank test.
**Supporting Figure 12**: Treatment stratification of DMFS and OS. **(A‐C)** Treatment stratification of DMFS in patients with the low‐risk (A), intermediate‐risk (B) and high‐risk scores (C). **(D‐F)** Treatment stratification of OS in patients with the low‐risk (D), intermediate‐risk (E) and high‐risk scores (F).

## Data Availability

The key raw data have been deposited at the Research Data Deposit public platform (RDDB2026157958) of Sun Yat‐sen University Cancer Center (www.researchdata.org.cn).
