# Supplementary material for: Circulating Microbial Metabolites Predict Tumor Relapse and Chemotherapy Efficacy in Nasopharyngeal Carcinoma
Source: MedComm (2020). 2026 Mar 18;7(4):e70687. doi: 10.1002/mco2.70687 (PMC13042733; doi:10.1002/mco2.70687)
Supplement: Supplementary file 1 — Supporting Table 1: Clinical characteristics of 48 paired patients with LA‐NPC experienced with or without tumor relapse in the discovery cohort. Supporting Table 2: Annotated differential metabolites in serum of 48 paired NPC patients with or without relapse in the discovery cohort. Supporting Table 3: A model of 4 metabolites with prognostic implication in the training cohort. Supporting Table 4: Multivariable Cox regression analysis of prognostic factors of patients in the training cohort. Supporting Table 5: Multivariable Cox regression analysis of prognostic factors of patients in the test cohort. Supporting Table 6: Multivariable Cox regression analysis of prognostic factors of patients in the validation cohort. Supporting Table 7: The C‐index of the nomogram and risk factors for prediction of disease‐free survival (DFS) in the training, test, and validation cohorts. Supporting Table 8: Clinical characteristics of patients with LA‐NPC in intermediate‐risk group experienced with or without IC. Supporting Table 9: Multivariable Cox regression analysis of prognostic factors of patients in the intermediate‐risk group. Supporting Table 10: Transparent reporting of a multivariable prediction model for individual prognosis or diagnosis statement (TRIPOD) Supporting Figure 1: Study population. Abbreviations: LA‐NPC, locoregional advanced nasopharyngeal carcinoma; TNM, tumor‐mode‐metastasis; LASSO, the least absolute shrinkage and selection operator. Supporting Figure 2: Characteristics of LA‐NPC patients in the discovery cohort. (A) Survival time of patients experienced with (n = 48) or without (n = 48) tumor relapse. We calculated the p values with Student t‐test. (B) Principal component analysis (PCA) plot showed the distribution of QC and samples. Supporting Figure 3: Differential serum metabolites in LA‐NPC patients with or without tumor relapse in the discovery cohort. The comparisons were conducted with Student t‐test. The center line represents the median norma [file MCO2-7-e70687-s001.docx]

**Circulating microbial metabolites predict tumor relapse and chemotherapy efficacy in nasopharyngeal carcinoma Authors:**

Jun-Yan Li^1, 2, #^, Yao Yao^1, 3, #^, Xi-Rong Tan^1,4, #^, Nan Si^1,3, #^, Wei Jiang^5,6, #^, Ying-Qi Lu^1,7^, Jia-Hao Dai^1,3^, Tian-Tian Yu^8^, Hao-Cheng Hu^9^, Yu-Fei Duan^1,3^, Sen-Yu Feng^1,7^, Sai-Wei Huang^1,7^, Ye-Lin Liang^1,7^, Sha Gong^1,3^, Na Liu^1,3,*^, Yu-Min Hu^1,8,*^, Ying-Qing Li^10,*^

**Affiliations:**

^1^ State Key Laboratory of Oncology in South China; Guangdong Key Laboratory of Nasopharyngeal Carcinoma Diagnosis and Therapy; Sun Yat-sen University Cancer Center, Guangzhou, People’s Republic of China

^2^ Department of Pathology, Sun Yat-sen University Cancer Center, Guangzhou, People’s Republic of China

^3^ Department of Experimental Research, Sun Yat-sen University Cancer Center, Guangzhou, People’s Republic of China

^4^ Department of Molecular Diagnosis, Sun Yat-sen University Cancer Center, Guangzhou, People’s Republic of China

^5^ Department of Radiation Oncology, Affiliated Hospital of Guilin Medical University, Guilin, People’s Republic of China

^6^ Key Laboratory of Oncology (Guilin Medical University), Education Department of Guangxi Zhuang Autonomous Region, Guilin, People’s Republic of China

^7^ Department of Radiation Oncology, Sun Yat-sen University Cancer Center, Guangzhou, People’s Republic of China

^8^ Metabolomics Research Center, Zhongshan School of Medicine, Sun Yat-sen University, Guangzhou, People’s Republic of China

^9^ School of Medicine, Shenzhen Campus of Sun Yat-sen University, Shenzhen, People’s Republic of China

^10^ Department of Outpatient, State Key Laboratory of Oncology in South China; Guangdong Key Laboratory of Nasopharyngeal Carcinoma Diagnosis and Therapy; Sun Yat-sen University Cancer Center, Guangzhou, People’s Republic of China

**^#^ Jun-Yan Li, Yao Yao, Xi-Rong Tan, Nan Si, and Wei Jiang contributed equally to this article.**

^*^ **Corresponding authors**

**Na Liu**, State Key Laboratory of Oncology in South China; Guangdong Key Laboratory of Nasopharyngeal Carcinoma Diagnosis and Therapy; Sun Yat-sen University Cancer Center, 651 Dongfeng Road East, Guangzhou 510060, People’s Republic of China; Telephone: +86-20-87342370; Fax: +86-20-87342370; E-mail: liun1@sysucc.org.cn

**Yu-Min Hu**, State Key Laboratory of Oncology in South China; Guangdong Key Laboratory of Nasopharyngeal Carcinoma Diagnosis and Therapy; Sun Yat-sen University Cancer Center, 651 Dongfeng Road East, Guangzhou 510060, People’s Republic of China; Telephone: +86-20-87343171; Fax: +86-20-87343171; E-mail: huym@sysucc.org.cn

**Ying-Qing Li**, Department of Outpatient, State Key Laboratory of Oncology in South China; Guangdong Key Laboratory of Nasopharyngeal Carcinoma Diagnosis and Therapy; Sun Yat-sen University Cancer Center, Guangzhou, People’s Republic of China; Telephone: +86-20-87342370; Fax: +86-20-87342370; E-mail: [liyingq1@sysucc.org.cn](mailto:liyingq1@sysucc.org.cn)

**Supplementary Table 1.** Clinical characteristics of 48 paired patients with LA-NPC experienced with or without tumor relapse in the discovery cohort.

|  | **Without Relapse**  **(%)** | **With Relapse**  **(%)** | ***P* value** |
| --- | --- | --- | --- |
| **Age (years old)** |  |  | 0.682 |
| < 45 | 23 (47.9%) | 21 (43.8%) |  |
| ≥ 45 | 25 (52.1%) | 27 (56.3%) |  |
| **Sex** |  |  | 0.789 |
| Female | 9 (18.8%) | 8 (16.7%) |  |
| Male | 39 (81.3%) | 40 (83.3%) |  |
| **T stage** |  |  | 0.807 |
| T1 | 2 (4.2%) | 1 (2.1%) |  |
| T2 | 5 (10.4%) | 3 (6.3%) |  |
| T3 | 26 (54.2%) | 27 (56.3%) |  |
| T4 | 15 (31.3%) | 17 (35.4%) |  |
| **N stage** |  |  | 0.780 |
| N0 | 1 (2.1%) | 1 (2.1%) |  |
| N1 | 19 (39.6%) | 15 (31.3%) |  |
| N2 | 11 (22.9%) | 15 (31.3%) |  |
| N3 | 17 (35.4%) | 17 (35.4%) |  |
| **TNM stage** |  |  | 0.529 |
| Ⅲ | 20 (41.7%) | 17 (35.4%) |  |
| Ⅳ | 28 (58.3%) | 31 (64.6%) |  |
| **EBV-DNA (copies/ml)** | |  | 0.637 |
| < 2000 | 13 (27.1%) | 11 (22.9%) |  |
| ≥ 2000 | 35 (72.9%) | 37 (77.1%) |  |

Note: TNM, tumor node metastasis; EBV, Epstein-Barr virus.

**Supplementary Table 2.** Annotated differential metabolites in serum of 48 paired NPC patients with or without relapse in the discovery cohort.

| **No.** | **HMDB ID** | **Name** | **Class** | ***P* value** | **Log2(FC)** |
| --- | --- | --- | --- | --- | --- |
| 1 | HMDB0000687 | L-Leucine | Amino acids | <0.001 | 0.68 |
| 2 | HMDB0001161 | gamma-Butyrobetaine @RT:8.023 | Fatty acids | <0.001 | 1.43 |
| 3 | HMDB0000300 | Uracil | Pyrimidines | <0.001 | -0.54 |
| 4 | HMDB0000162 | L-Proline | Amino acid | 0.001 | 0.46 |
| 5 | HMDB0000895 | Acetylcholine | Others | 0.001 | 1.67 |
| 6 | HMDB0002712 | 1,5-Anhydro-D-glucitol | Carbohydrates | 0.003 | -0.39 |
| 7 | HMDB0002721 | 1-Methylinosine | Nucleosides | 0.009 | 0.45 |
| 8 | HMDB0000226 | Orotic acid | Pyrimidines | 0.009 | -0.60 |
| 9 | HMDB0029200 | Ferulic acid 4-sulfate | Others | 0.011 | -0.87 |
| 10 | HMDB0000497 | Dihydrouridine | Carbohydrates | 0.011 | 0.39 |
| 11 | HMDB0000630 | Cytosine | Pyrimidines | 0.012 | 0.46 |
| 12 | HMDB0001161 | gamma-Butyrobetaine @RT:0.803 | Fatty acids | 0.012 | 0.50 |
| 13 | HMDB0003681 | 4-Acetamidobutanoic acid | Amino acids | 0.013 | 0.69 |
| 14 | HMDB0060460 | cis-4-Hydroxy-D-proline | Amino acids | 0.014 | 0.77 |
| 15 | HMDB0006344 | N-Phenylacetylglutamine | Amino acids | 0.026 | 0.71 |
| 16 | HMDB0002894 | 5-Methylcytosine | Pyrimidines | 0.029 | 0.33 |
| 17 | HMDB0000641 | L-Glutamine | Amino acids | 0.029 | 0.28 |
| 18 | HMDB0012150 | 6-Acetamido-2-oxohexanoic acid | Organic acids | 0.036 | -0.51 |
| 19 | HMDB0254310 | Maleamic acid | Fatty acids | 0.039 | -0.29 |
| 20 | HMDB0011757 | N-Acetylvaline | Amino acids | 0.040 | 1.45 |
| 21 | HMDB0012308 | Vanillin | Benzenoids | 0.042 | -0.43 |
| 22 | HMDB0062188 | 1-Nitrosonaphthalene | Benzenoids | 0.044 | 0.35 |
| 23 | HMDB0002205 | L-Homocysteic acid | Amino acids | 0.049 | -0.35 |
| 24 | HMDB0001138 | N-Acetylglutamic acid | Amino acids | 0.049 | 0.75 |

Footnote: FC, Fold Change.

**Supplementary Table 3.** A model of 4 metabolites with prognostic implication in the training cohort.

| **Metabolite** | **LASSO coefficients** | **Relative abundance** | |
| --- | --- | --- | --- |
|  |  | **median** | **range** |
| 4-Acetamidobutanoic acid | 0.176 | 0.872 | 0.040-5.163 |
| L-Proline | 0.185 | 0.890 | 0.017-2.327 |
| N-Phenylacetylglutamine | 0.257 | 0.669 | 0.019-5.691 |
| Vanillin | -0.107 | 0.509 | 0.010-6.139 |

Note: LASSO, the least absolute shrinkage and selection operator.

**Supplementary Table 4.** Multivariable Cox regression analysis of prognostic factors of patients in the training cohort.

|  | **Multivariate analysis** | | |
| --- | --- | --- | --- |
| **Variable** | **HR** | **95%CI** | ***P* value** |
| **Disease-free survival** |  |  |  |
| N stage | 2.17 | 1.26-3.74 | 0.005 |
| EBV-DNA | 2.46 | 1.36-4.46 | 0.003 |
| Metabolite risk model |  |  |  |
| *Median- vs. Low- risk* | 4.74 | 2.28-9.87 | <0.001 |
| *High- vs. Low- risk* | 14.74 | 6.10-35.64 | <0.001 |
| Induction chemotherapy | 0.39 | 0.20-0.73 | 0.003 |
| **Distant metastasis-free survival** |  |  |  |
| N stage | 2.16 | 1.03-4.50 | 0.041 |
| EBV-DNA | 3.48 | 1.44-8.42 | 0.006 |
| Metabolite risk model |  |  |  |
| *Median- vs. Low- risk* | 3.00 | 1.18-7.66 | 0.022 |
| *High- vs. Low- risk* | 13.96 | 4.72-41.28 | <0.001 |
| Induction chemotherapy | 0.35 | 0.14-0.88 | 0.025 |
| **Overall survival** |  |  |  |
| N stage | 2.01 | 1.08-3.74 | 0.027 |
| EBV-DNA | 3.15 | 1.53-6.47 | 0.002 |
| Metabolite risk model |  |  |  |
| *Median- vs. Low- risk* | 3.69 | 1.67-8.19 | 0.001 |
| *High- vs. Low- risk* | 16.36 | 6.51-41.10 | <0.001 |
| Induction chemotherapy | 0.36 | 0.17-0.75 | 0.007 |

Note: We calculated hazard ratio (HRs) and *P* values by adjusted multivariable Cox regression model, including age (≥45 vs. <45 years old), sex (male vs. female), WHO pathological type (undifferentiated vs. Others), T stage (T3-4 vs. T1-2), N stage (N2-3 vs. N0-1), EBV-DNA (≥2000 vs. <2000 copies/ml), metabolite risk model (High-/Median- vs. Low- risk), and induction chemotherapy (no vs. yes) as covariates. Only variables that were significantly associated with survival (*P*<0.05) are presented. EBV, Epstein-Barr virus.

**Supplementary Table 5.** Multivariable Cox regression analysis of prognostic factors of patients in the test cohort.

|  | **Multivariate analysis** | | |
| --- | --- | --- | --- |
| **Variable** | **HR** | **95%CI** | ***P* value** |
| **Disease-free survival** |  |  |  |
| EBV-DNA | 2.02 | 1.03-3.94 | 0.040 |
| Metabolite risk model |  |  |  |
| *Median- vs. Low- risk* | 3.90 | 2.00-7.58 | <0.001 |
| *High- vs. Low- risk* | 7.89 | 3.35-18.59 | <0.001 |
| **Distant metastasis-free survival** |  |  |  |
| N stage | 2.37 | 1.08-5.21 | 0.032 |
| EBV-DNA | 2.74 | 1.10-6.82 | 0.031 |
| Metabolite risk model |  |  |  |
| *Median- vs. Low- risk* | 2.62 | 1.19-5.80 | 0.017 |
| *High- vs. Low- risk* | 4.89 | 1.77-13.46 | 0.002 |
| Induction chemotherapy | 0.43 | 0.21-0.88 | 0.021 |
| **Overall survival** |  |  |  |
| Age | 2.77 | 1.33-5.78 | 0.007 |
| Metabolite risk model |  |  |  |
| *Median- vs. Low- risk* | 5.81 | 2.31-14.63 | <0.001 |
| *High- vs. Low- risk* | 13.67 | 4.59-40.67 | <0.001 |

Note: We calculated hazard ratio (HRs) and *P* values by adjusted multivariale Cox regression model, including age (≥45 vs. <45 years old), sex (male vs. female), WHO pathological type (undifferentiated vs. Others), T stage (T3-4 vs. T1-2), N stage (N2-3 vs. N0-1), EBV-DNA (≥2000 vs. <2000 copies/ml), metabolite risk model (High-/Median- vs. Low- risk), and induction chemotherapy (no vs. yes) as covariates. Only variables that were significantly associated with survival (*P*<0.05) are presented. EBV, Epstein-Barr virus.

**Supplementary Table 6.** Multivariable Cox regression analysis of prognostic factors of patients in the validation cohort.

|  | **Multivariate analysis** | | |
| --- | --- | --- | --- |
| **Variable** | **HR** | **95%CI** | ***P* value** |
| **Disease-free survival** |  |  |  |
| N stage | 3.66 | 1.13-11.80 | 0.030 |
| Metabolite risk model |  |  |  |
| *Median- vs. Low- risk* | 5.21 | 2.50-10.88 | <0.001 |
| *High- vs. Low- risk* | 17.35 | 6.79-44.37 | <0.001 |
| **Distant metastasis-free survival** |  |  |  |
| Metabolite risk model |  |  |  |
| *Median- vs. Low- risk* | 5.88 | 2.22-15.55 | <0.001 |
| *High- vs. Low- risk* | 18.77 | 5.67-62.14 | <0.001 |
| **Overall survival** |  |  |  |
| Metabolite risk model |  |  |  |
| *Median- vs. Low- risk* | 4.45 | 1.24-15.98 | 0.022 |
| *High- vs. Low- risk* | 16.37 | 3.65-73.52 | <0.001 |

Note: We calculated hazard ratio (HRs) and *P* values by adjusted multivariale Cox regression model, including age (≥45 vs. <45 years old), sex (male vs. female), WHO pathological type (undifferentiated vs. Others), T stage (T3-4 vs. T1-2), N stage (N2-3 vs. N0-1), metabolite risk model (High-/Median- vs. Low- risk), and induction chemotherapy (no vs. yes) as covariates. Only variables that were significantly associated with survival (*P*<0.05) are presented. EBV, Epstein-Barr virus.

**Supplementary Table 7.** The C-index of the nomogram and risk factors for prediction of disease-free survival (DFS) in the training, test, and validation cohorts.

|  | **C-index** | **95% CI** |
| --- | --- | --- |
| **Training cohort** |  |  |
| N stage | 0.597 | 0.535-0.659 |
| EBV-DNA | 0.584 | 0.524-0.643 |
| Risk model | 0.724 | 0.664-0.784 |
| Nomogram | 0.774 | 0.720-0.828 |
| **Test cohort** |  |  |
| N stage | 0.597 | 0.529-0.666 |
| EBV-DNA | 0.611 | 0.550-0.673 |
| Risk model | 0.724 | 0.658-0.789 |
| Nomogram | 0.764 | 0.701-0.828 |
| **Validation cohort** |  |  |
| N stage | 0.561 | 0.519-0.603 |
| Risk model | 0.712 | 0.647-0.778 |
| Nomogram | 0.731 | 0.668-0.795 |

Note: C-index, concordance index; EBV, Epstein-Barr virus.

**Supplementary Table 8.** Clinical characteristics of patients with LA-NPC in intermediate-risk group experienced with or without IC.

|  | Without IC  (%) | With IC  (%) | *P* value |
| --- | --- | --- | --- |
| **Age (years old)** |  |  | 0.603 |
| < 45 | 45 (51.1) | 36 (55.4) |  |
| ≥ 45 | 43 (48.9) | 29 (44.6) |  |
| **Sex** |  |  | 0.483 |
| Female | 15 (17.0) | 14 (21.5) |  |
| Male | 73 (83.0) | 51 (78.5) |  |
| **T stage** |  |  | 0.770 |
| T1 | 3 (3.4) | 4 (6.2) |  |
| T2 | 8 (9.1) | 4 (6.2) |  |
| T3 | 52 (59.1) | 37 (56.9) |  |
| T4 | 25 (28.4) | 20 (30.8) |  |
| **N stage** |  |  | 0.348 |
| N0 | 5 (5.7) | 1 (1.5) |  |
| N1 | 43 (48.9) | 27 (41.5) |  |
| N2 | 27 (30.7) | 23 (35.4) |  |
| N3 | 13 (14.8) | 14 (21.5) |  |
| **TNM stage** |  |  | 0.306 |
| Ⅲ | 52 (59.1) | 33 (50.8) |  |
| Ⅳ | 36 (40.9) | 32 (49.2) |  |
| **EBV-DNA (copies/ml)** | |  | 0.303 |
| < 2000 | 37 (42.0) | 22 (33.8) |  |
| ≥ 2000 | 51 (58.0) | 43 (66.2) |  |

Note: TNM, tumor node metastasis; EBV, Epstein-Barr virus.

**Supplementary Table 9.** Multivariable Cox regression analysis of prognostic factors of patients in the intermediate-risk group.

|  | **Multivariate analysis** | | |
| --- | --- | --- | --- |
| **Variable** | **HR** | **95%CI** | ***P* value** |
| **Disease-free survival** |  |  |  |
| N stage | 1.67 | 1.01-2.77 | 0.046 |
| EBV-DNA | 1.93 | 1.12-3.33 | 0.019 |
| Induction chemotherapy | 0.38 | 0.22-0.66 | 0.001 |
| **Distant metastasis-free survival** |  |  |  |
| N stage | 2.31 | 1.11-4.81 | 0.026 |
| Induction chemotherapy | 0.29 | 0.13-0.65 | 0.003 |
| **Overall survival** |  |  |  |
| N stage | 1.99 | 1.07-3.70 | 0.029 |
| EBV-DNA | 2.22 | 1.10-4.49 | 0.026 |
| Induction chemotherapy | 0.32 | 0.16-0.63 | 0.001 |

Note: We calculated hazard ratio (HRs) and *P* values by adjusted multivariable Cox regression model, including age (≥45 vs. <45 years old), sex (male vs. female), WHO pathological type (undifferentiated vs. Others), T stage (T3-4 vs. T1-2), N stage (N2-3 vs. N0-1), EBV-DNA (≥2000 vs. <2000 copies/ml), and induction chemotherapy (no vs. yes) as covariates. Only variables that were significantly associated with survival (*P*<0.05) are presented. EBV, Epstein-Barr virus.

**Supplementary Table 10.** Transparent reporting of a multivariable prediction model for individual prognosis or diagnosis statement (TRIPOD).

| **Section/Topic** | **Item** | **Checklist Item** | **Page** |
| --- | --- | --- | --- |
| **Title and abstract** | | | |
| Title | 1 | Identify the study as developing and/or validating a multivariable prediction model, the target population, and the outcome to be predicted. | 1 |
| Abstract | 2 | Provide a summary of objectives, study design, setting, participants, sample size, predictors, outcome, statistical analysis, results, and conclusions. | 3 |
| **Introduction** | | | |
| Background and objectives | 3a | Explain the medical context (including whether diagnostic or prognostic) and rationale for developing or validating the multivariable prediction model, including references to existing models. | 4-5 |
|  | 3b | Specify the objectives, including whether the study describes the development or validation of the model or both. | 5 |
| **Methods** | | | |
| Source of data | 4a | Describe the study design or source of data (e.g., randomized trial, cohort, or registry data), separately for the development and validation data sets, if applicable. | 14 |
|  | 4b | Specify the key study dates, including start of accrual; end of accrual; and, if applicable, end of follow-up. | 14 |
| Participants | 5a | Specify key elements of the study setting (e.g., primary care, secondary care, general population) including number and location of centres. | 14 |
|  | 5b | Describe eligibility criteria for participants. | 14 |
|  | 5c | Give details of treatments received, if relevant. | 14 |
| Outcome | 6a | Clearly define the outcome that is predicted by the prediction model, including how and when assessed. | 17 |
|  | 6b | Report any actions to blind assessment of the outcome to be predicted. | Not applicable |
| Predictors | 7a | Clearly define all predictors used in developing or validating the multivariable prediction model, including how and when they were measured. | 15-17 |
|  | 7b | Report any actions to blind assessment of predictors for the outcome and other predictors. | Not applicable |
| Sample size | 8 | Explain how the study size was arrived at. | 15 |
| Missing data | 9 | Describe how missing data were handled (e.g., complete-case analysis, single imputation, multiple imputation) with details of any imputation method. | 16 |
| Statistical analysis methods | 10c | For validation, describe how the predictions were calculated. | 7-8 |
|  | 10d | Specify all measures used to assess model performance and, if relevant, to compare multiple models. | 8-9 |
|  | 10e | Describe any model updating (e.g., recalibration) arising from the validation, if done. | Not applicable |
| Risk groups | 11 | Provide details on how risk groups were created, if done. | 7 |
| Development vs. validation | 12 | For validation, identify any differences from the development data in setting, eligibility criteria, outcome, and predictors. | Table 1 |
| **Results** | | | |
| Participants | 13a | Describe the flow of participants through the study, including the number of participants with and without the outcome and, if applicable, a summary of the follow-up time. A diagram may be helpful. | Table 1  Figure S1 |
|  | 13b | Describe the characteristics of the participants (basic demographics, clinical features, available predictors), including the number of participants with missing data for predictors and outcome. | Table 1 |
|  | 13c | For validation, show a comparison with the development data of the distribution of important variables (demographics, predictors and outcome). | Table 1 |
| Model performance | 16 | Report performance measures (with CIs) for the prediction model. | 7-10 |
| Model-updating | 17 | If done, report the results from any model updating (i.e., model specification, model performance). | Not applicable |
| **Discussion** | | | |
| Limitations | 18 | Discuss any limitations of the study (such as nonrepresentative sample, few events per predictor, missing data). | 13 |
| Interpretation | 19a | For validation, discuss the results with reference to performance in the development data, and any other validation data. | 11-13 |
|  | 19b | Give an overall interpretation of the results, considering objectives, limitations, results from similar studies, and other relevant evidence. | 11-13 |
| Implications | 20 | Discuss the potential clinical use of the model and implications for future research. | 11-12 |
| **Other information** | | | |
| Supplementary information | 21 | Provide information about the availability of supplementary resources, such as study protocol, Web calculator, and data sets. | 19 |
| Funding | 22 | Give the source of funding and the role of the funders for the present study. | 19-20 |


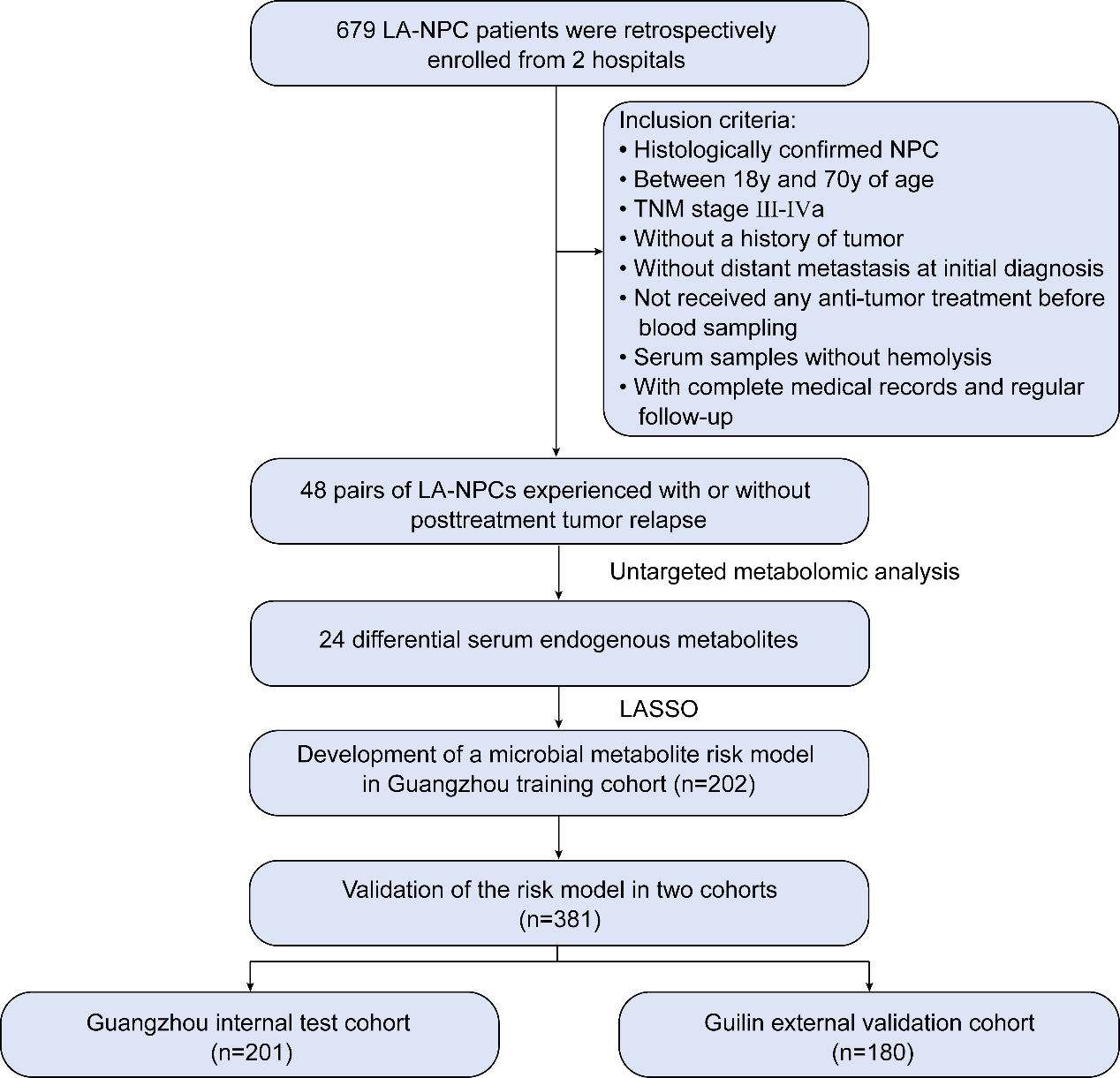


**Supplementary Figure 1.** **Study population.**

Abbreviations: LA-NPC, locoregional advanced nasopharyngeal carcinoma; TNM, tumor-mode-metastasis; LASSO, the least absolute shrinkage and selection operator.

**
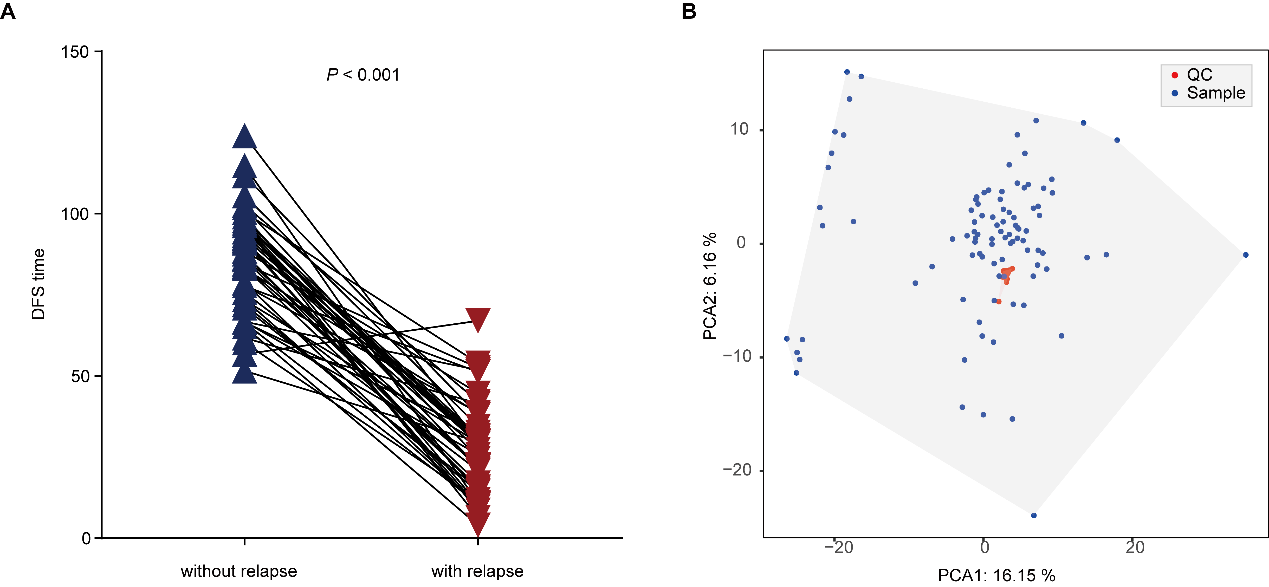
**

**Supplementary Figure 2.** **Characteristics of LA-NPC patients in the discovery cohort.**

**(A)** Survival time of patients experienced with (n=48) or without (n=48) tumor relapse. We calculated the P values with Student t-test. **(B)** Principal component analysis (PCA) plot showed the distribution of QC and samples.

**
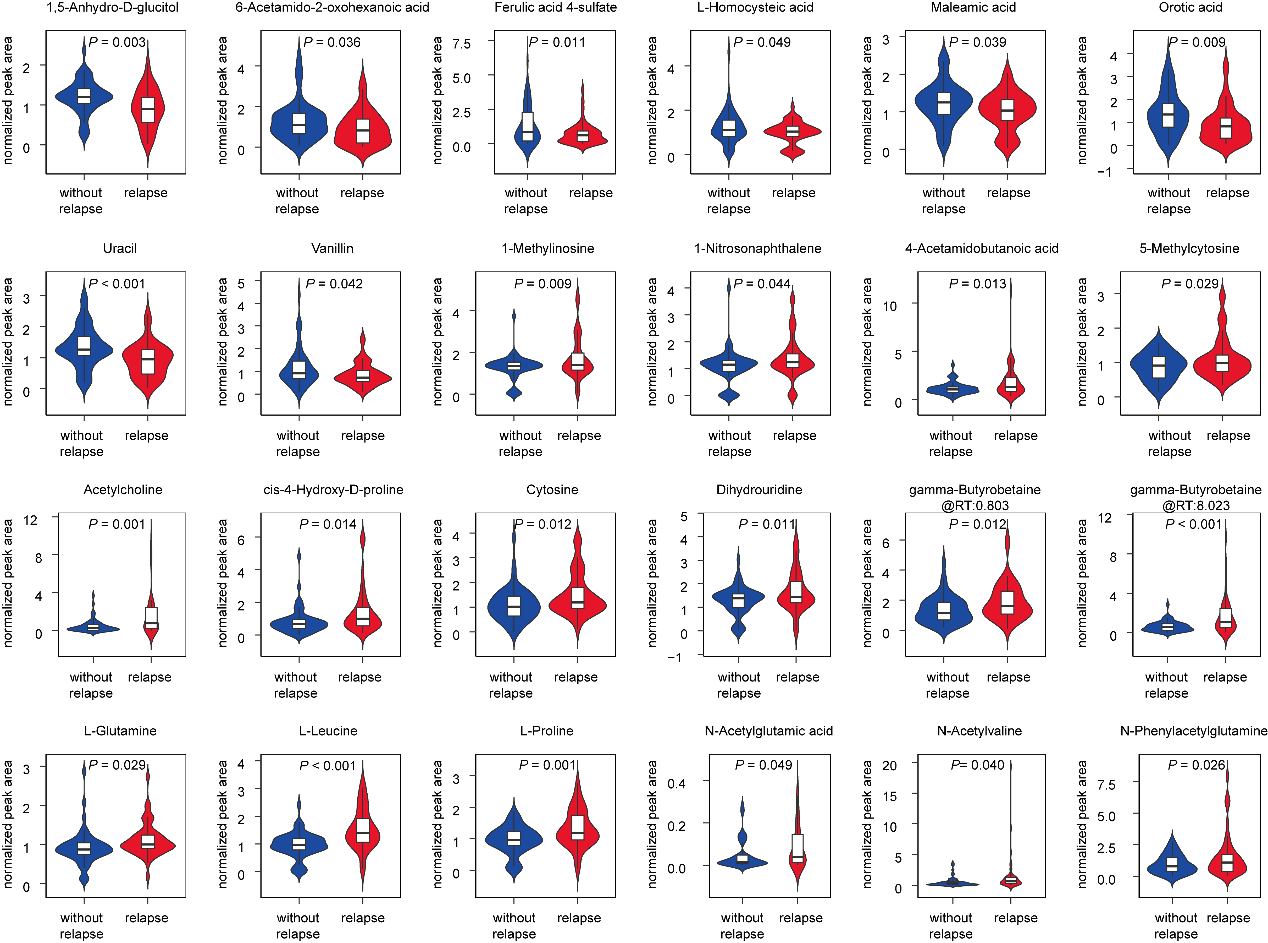
**

**Supplementary Figure 3.** **Differential serum metabolites in LA-NPC patients with or without tumor relapse in the discovery cohort.** The comparisons were conducted with Student t-test. The center line represents the median normalized abundance, and the box bound represents the inter-quartile range.

**
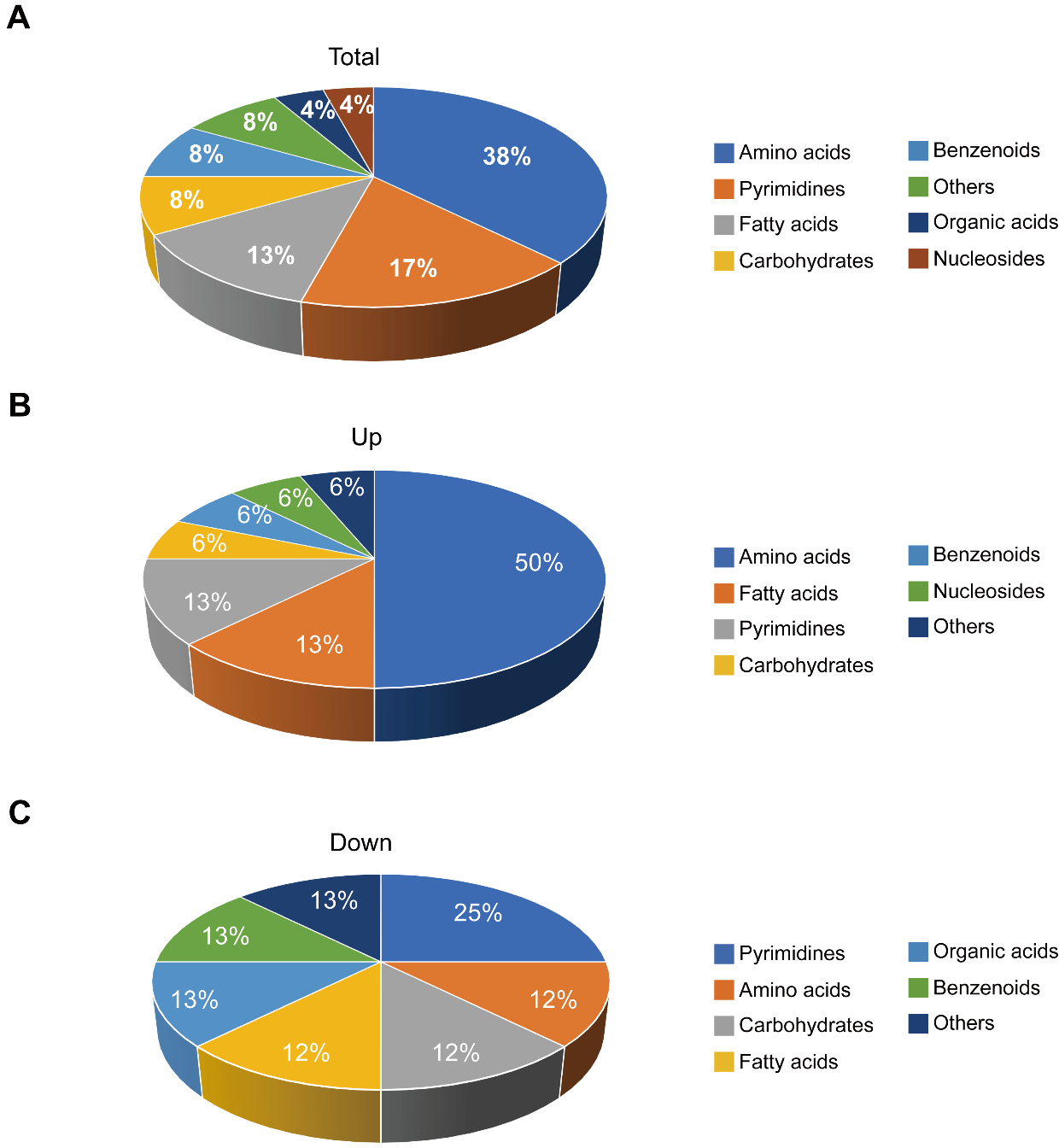
**

**Supplementary Figure 4.** **The category percentage of differential metabolites.**

**(A)** The categories of the total differential metabolites, **(B)** enriched metabolites and **(C)** diminished metabolites in LA-NPC patients with or without tumor relapse.


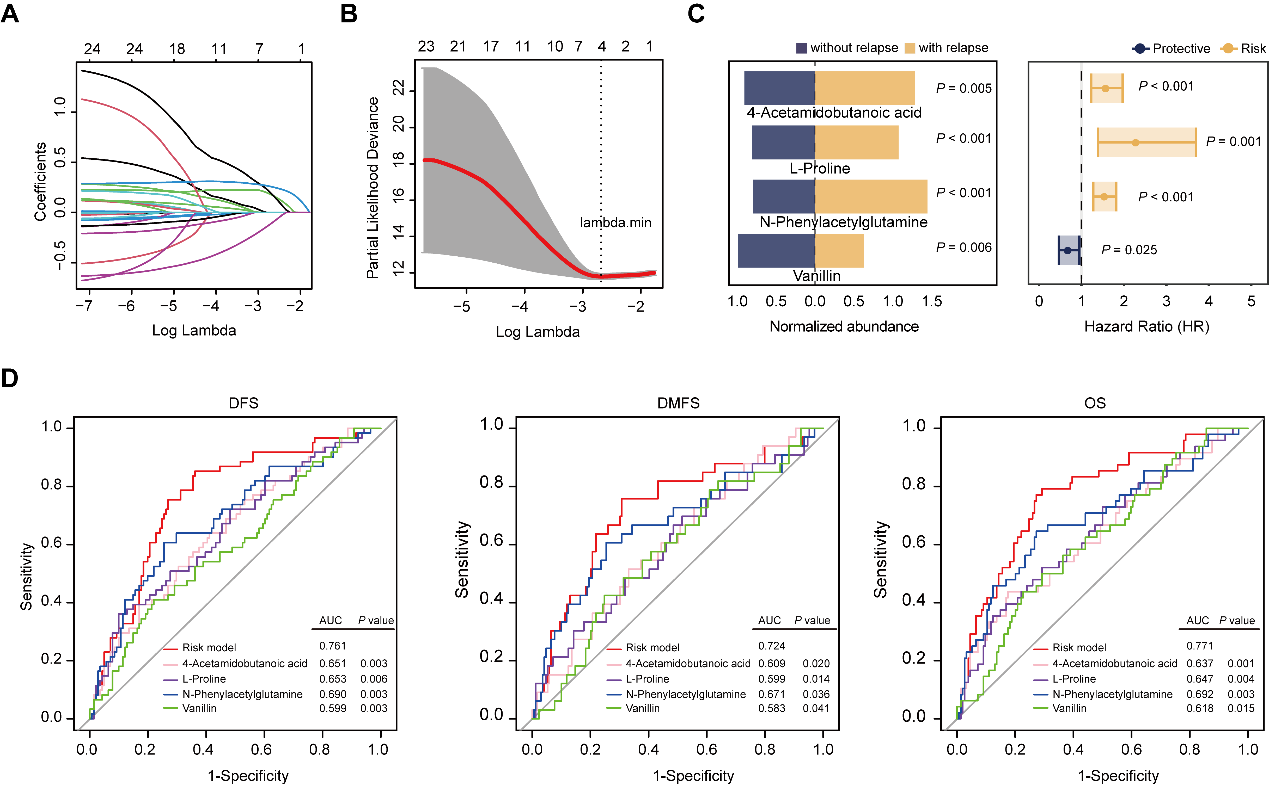


**Supplementary Figure 5.** **Construction of the microbial metabolite risk model in the training cohort.**

**(A)** LASSO coefficient profiles of the candidate metabolites for model construction. **(B)** Ten-time cross-validations in LASSO were performed to tune the parameter selection. Dotted vertical lines are drawn at the optimal values by minimum criteria. **(C)** Left panel: Comparison of the normalized abundance of four metabolites identified by LASSO. We calculated the P values with Student t-test. Right panel: Forest plot of the four metabolites by univariate Cox regression analysis. **(D)** ROC analysis compared the risk model with each single risk metabolite for disease-free survival (DFS), distant metastasis-free survival (DMFS), and overall survival (OS).


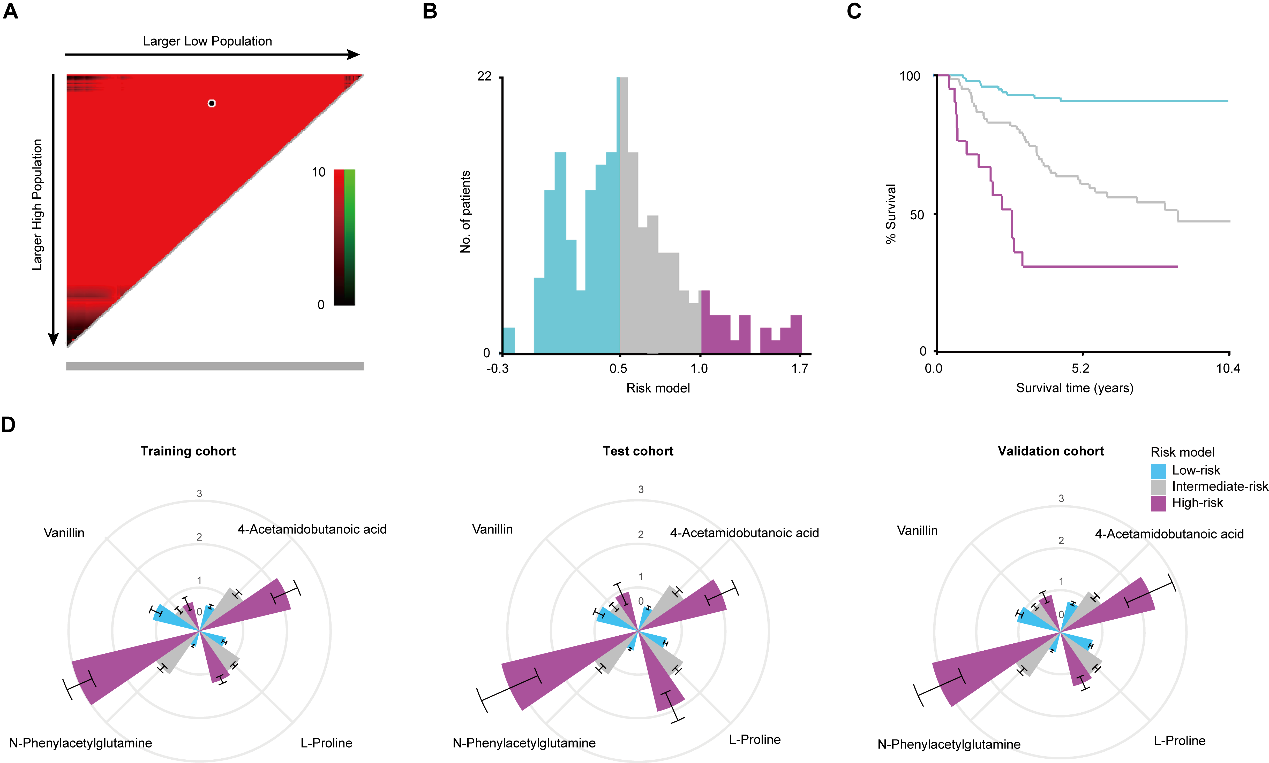


**Supplementary Figure 6.** **Determination of optimum cutoff points for microbial metabolite risk model.**

**(A-C)** Thresholds selection using X-tile in the training cohort. **(D)** Bar plots showed the change trends of four risk metabolites determined by the risk model in the training cohort (left), test cohort (median) and validation cohort (right).


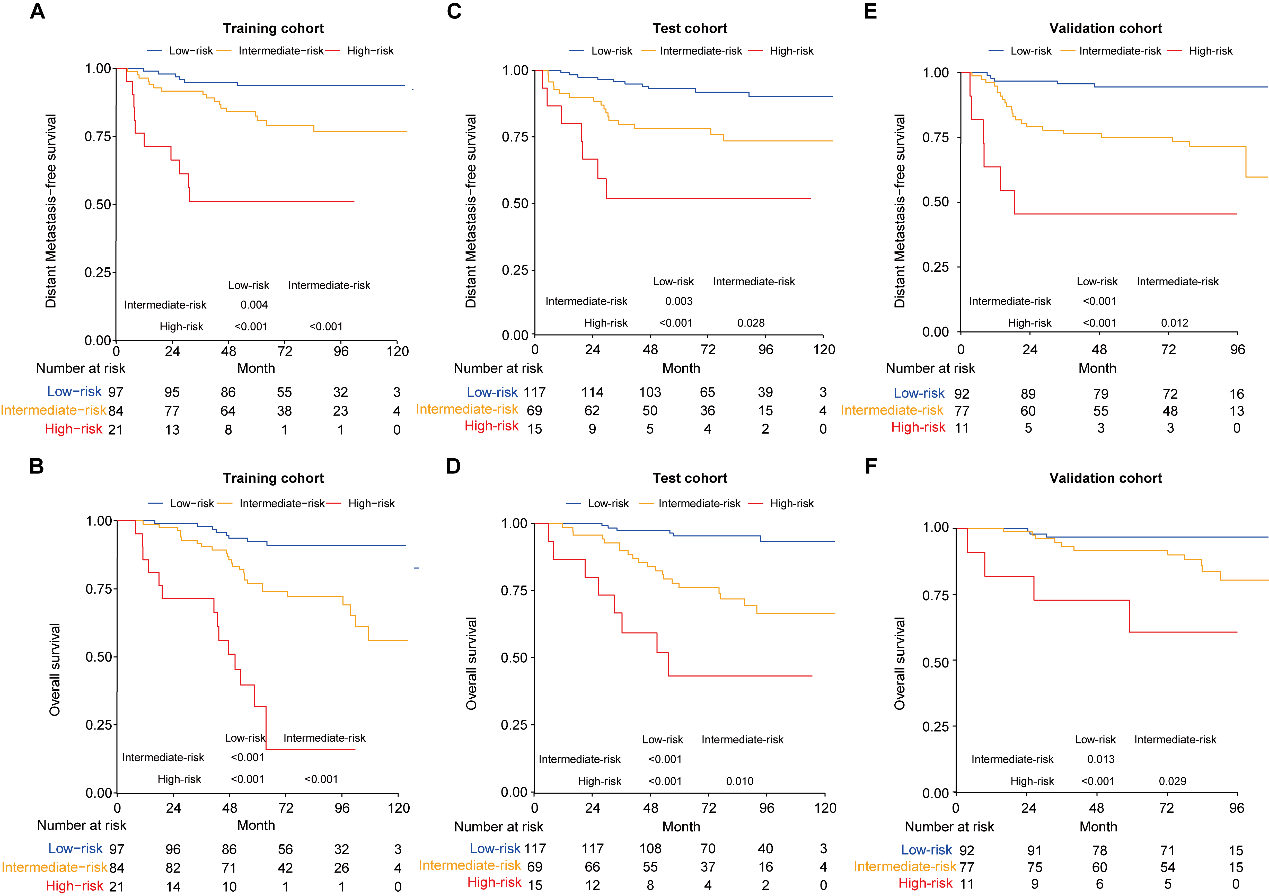


**Supplementary Figure 7.** Kaplan-Meier curves of DMFS and OS in the training, test and validation cohorts according to the microbial metabolite risk model.

**(A-B)** Kaplan-Meier curves of DMFS (A) and OS (B) in the training cohort by the risk model. **(C-D)** Kaplan-Meier curves of DMFS (C) and OS (D) in the test cohort by the risk model. **(E-F)** Kaplan-Meier curves of DMFS (E) and OS (F) in the validation cohort by the risk model. We calculated the P values with unadjusted log-rank test.


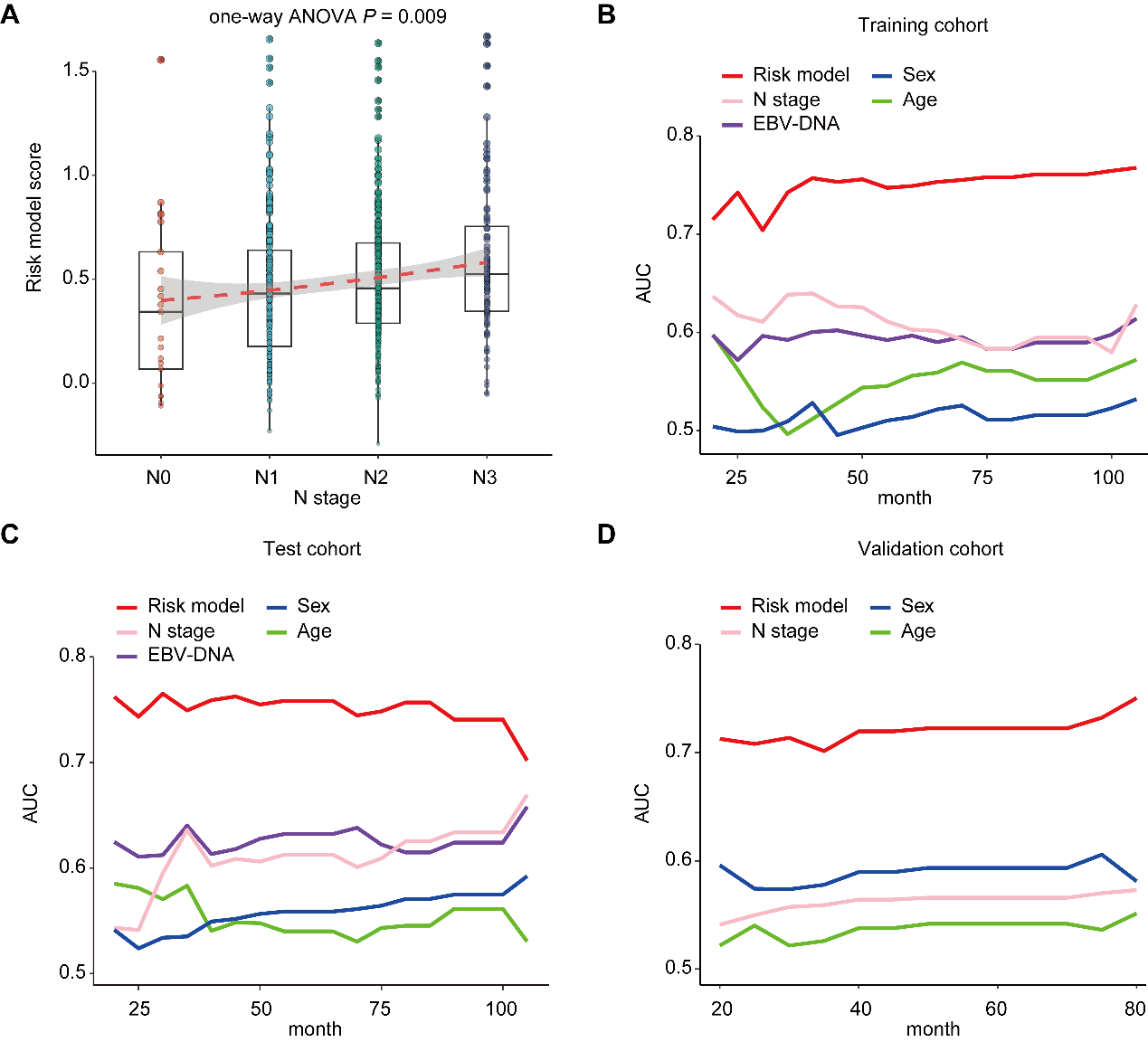


**Supplementary Figure 8.** Prognostic performance of the microbial metabolite risk model and clinical characteristics.

**(A)** Boxplots represented the risk model score determined by N stage. **(B-D)** Time-dependent AUC analysis compared the microbial metabolite risk model with clinical characteristics for DFS in a continuous period in the training cohort (B), test cohort (C), and validation cohort (D).


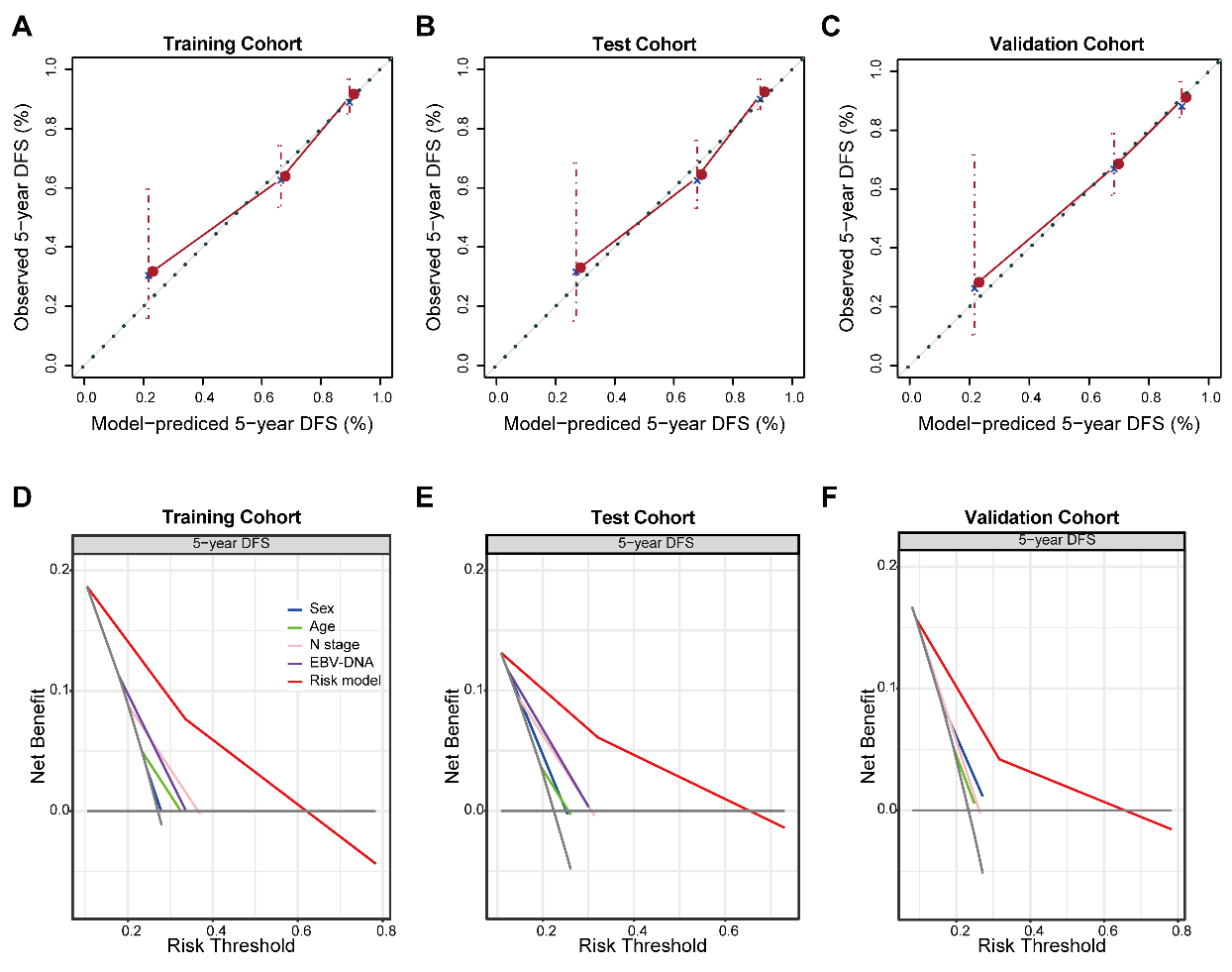


**Supplementary Figure 9.** Calibration curve (**A-C**) and decision curve analysis (**D-E**) of the metabolite risk model in training, test, and validation cohorts.


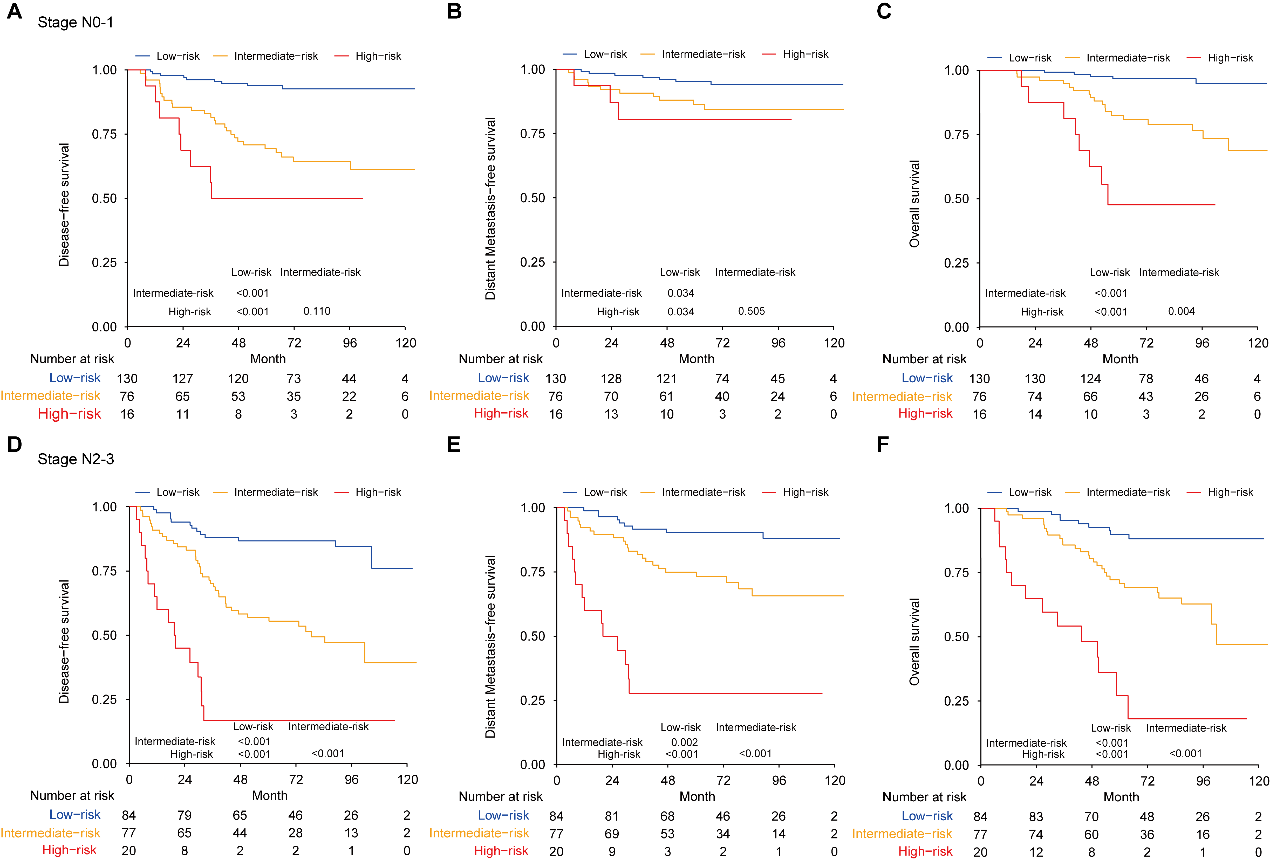


**Supplementary Figure 10.** Kaplan-Meier curves of DFS, DMFS and OS according to the microbial metabolite risk model stratified by N stage.

**(A-C)** DFS (A), DMFS (B) and OS (C) in the stage N0-1 group according to the microbial metabolite risk model. **(D-E)** DFS (D), DMFS (E) and OS (F) in the stage N2-3 group according to the microbial metabolite risk model. We calculated the *P* values with unadjusted long-rank test.


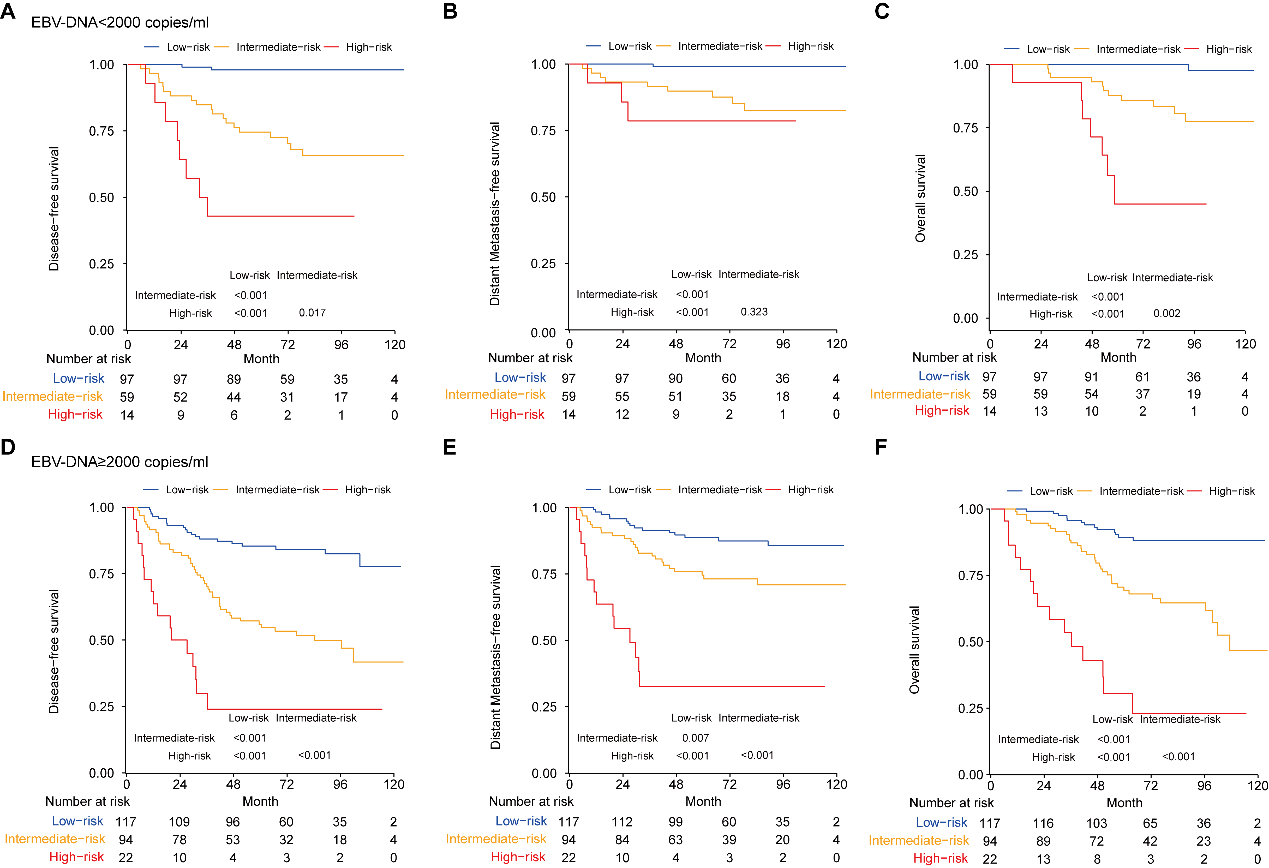


**Supplementary Figure 11.** Kaplan-Meier curves of DFS, DMFS and OS according to the microbial metabolite risk model stratified by plasma EBV-DNA load.

**(A-C)** DFS (A), DMFS (B) and OS (C) in the low plasma EBV-DNA load group (<2000 copies/ml) according to the microbial metabolite risk model. **(D-E)** DFS (D), DMFS (E) and OS (F) in the high plasma EBV-DNA load group (≥2000 copies/ml) according to the microbial metabolite risk model. We calculated the *P* values with unadjusted long-rank test.


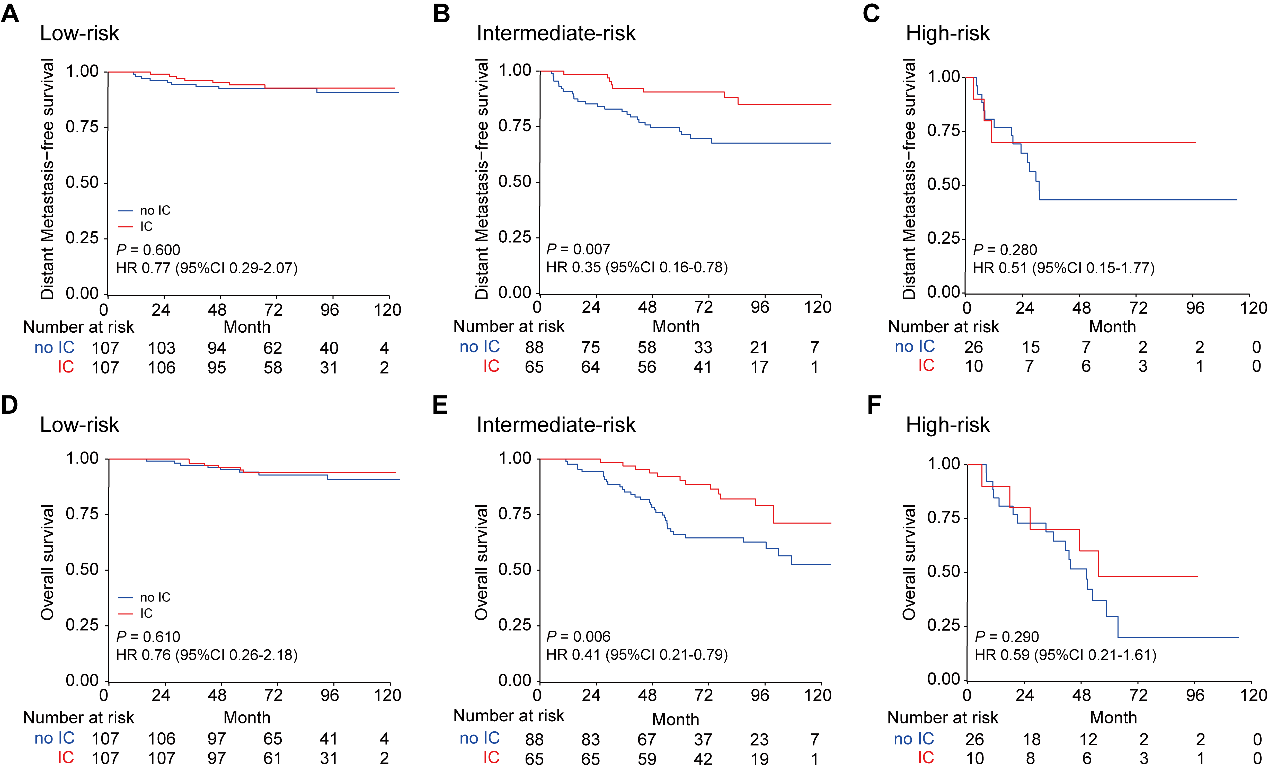


**Supplementary Figure 12.** Treatment stratification of DMFS and OS.

**(A-C)** Treatment stratification of DMFS in patients with the low-risk (A), intermediate-risk (B) and high-risk scores (C). **(D-F)** Treatment stratification of OS in patients with the low-risk (D), intermediate-risk (E) and high-risk scores (F).
